# A systematic review and meta-analysis of the impact of relaxation techniques to reduce burden of disease in patients with psychotic disorders

**DOI:** 10.1038/s41598-026-44310-0

**Published:** 2026-03-24

**Authors:** Nina Schlößer, Christian Theisen, Eva Meisenzahl, Carolin Kieckhäfer

**Affiliations:** Department of Psychiatry and Psychotherapy, Medical Faculty, Heinrich- Heine University, Düsseldorf, Germany

**Keywords:** Relaxation, Schizophrenia, Psychosis, Mindfulness, Stress reduction, Diseases, Health care, Medical research, Psychology, Psychology

## Abstract

The role of psychosocial stress as a factor in both the development of psychosis and the exacerbation of acute psychotic episodes is increasingly well understood. Various approaches to stress reduction have been shown to be helpful for psychosis patients, but they are rarely included in guidelines and treatment algorithms. In this systematic review and meta-analysis, we examined the effectiveness and potential adverse effects of interventions that specifically aim to reduce stress in psychosis patients. To do so, we systematically searched five databases (PubMed, Scopus, Web of Science, PsycINFO, Cochrane-Library) for studies on relaxation interventions in psychosis treatment. Of the identified studies, 24 (*N* = 1292 patients) were eligible for inclusion. We performed a systematic review and general meta-analysis and a subgroup analysis for patient setting (inpatient, outpatient, rehabilitative, or mixed). The general analysis showed small to moderate effects of the interventions on general, positive, and negative symptoms but not for the reduction of stress. In the subgroup analysis, psychosis patients in outpatient and rehabilitative settings benefitted more from the interventions than those in an inpatient setting. No adverse effects were reported. These results help to consider the integration of relaxation techniques for stress reduction as an add-on therapy in psychosis treatment.

**Supplementary Information:**

The online version contains supplementary material available at 10.1038/s41598-026-44310-0.

## Introduction

Psychotic disorders represent a heterogeneous group of conditions, including schizophrenia, schizoaffective disorder, first-episode psychosis, and transient psychotic disorders, and are associated with significant impairment and burden^1,2^. The clinical presentation of psychotic disorders in general includes positive, negative, and affective symptoms, cognitive impairment, and disorganized thinking and behavior^3,4^. Furthermore, many patients suffer of psychiatric comorbidities, such as substance use, panic disorders, obsessive-impulse disorder, posttraumatic stress disorder, and depression^5,6^; as well as somatic comorbidities, such as cardiovascular diseases, type 2 diabetes and chronic obstructive pulmonary disease^7^. In addition, patients suffer an elevated risk of suicide^6,8^. The somatic comorbidities, side effects and suicide risk reduce the life expectancy of schizophrenia patients and patients with psychotic disorders by 12–15 years^9,10^. Furthermore, only a small proportion of patients meet the criteria for recovery^11,12^.

The etiology of psychosis is multicausal^13,14^, and includes the processing of stress^15^. The influence of psychosocial stress on the development of psychosis has been highlighted and investigated for centuries^16^. Stress refers to psychosocial adversities such as acute life events, chronic social adversity, or traumatic experiences^17^. Literature emphasises a direct interaction between the exposure to psychosocial stress and an increase of striatal dopamine release in prone individuals with a dysregulated HPA axis^17^. Alterations in the dopaminergic pathway are known to be linked directly to positive as well as negative symptoms of psychosis patients.

Hence, psychoeducation, psychotherapy and even mindfulness-based therapy have found their way into the treatment of psychosis patients to improve patients’ coping strategies and stress management skills. However, international guidelines do not recommend stress reduction as intervention explicitly although arousal and stress during psychotic episodes often lead to difficulties with treatment and increase the likelihood that coercive measures are needed.

Clinicians tend to be reluctant to use relaxation interventions because of a few case reports in which *intense* meditation showed a close temporal correlation to psychotic exacerbations^18–20^. Nevertheless, a growing body of reviews highlights the positive effects of meditation and mindfulness in psychosis patients^21^. Mindfulness-based interventions are rooted in Eastern meditation practices and refer to a state of being in the moment^22^. They cover a wide range of practices and programs and have been shown to help improve general psychopathology in adults with psychosis^23^. Several reviews and meta-analyses that specifically evaluated the effects of mindfulness interventions on psychotic symptoms found that the interventions lead to a reduction of both positive^24–26^ and negative symptoms^23,25,27^. Furthermore, the analyses by Khoury et al. (2013) and Yip et al. (2022) showed a significant positive effect of mindfulness on the quality of life of schizophrenia patients.

Mindfulness-based interventions, which include techniques such as yoga and body scanning, represent a large share of relaxation techniques. However, these interventions also include other interventions, e.g., the mind-body interventions tai chi and qigong, which both include movement sequences, breathing techniques, and mindfulness^28^. Although mindfulness is a part of tai chi and qigong, the former showed only inconsistent effects on schizophrenia symptoms^29^ and the latter did not show any effect at all^30^. Another relaxation intervention is autogenic training, which is based on autosuggestion and was established in the 1930s in Germany; however, studies on its usefulness in schizophrenia show inconsistent effects^31^. In contrast, progressive muscle relaxation, a method where individuals first tense and then relax a muscle group for several seconds^32^, showed robust results^33^, and initial results on the use of biofeedback, which gives patients direct or transformed feedback on biological information from their body, were also promising^34,35^.

As relaxation techniques are not currently recommended in most clinical guidelines^36,37^ despite the above-mentioned positive results, we performed a meta-analysis with the aim to collate the current evidence from randomized controlled studies on various relaxation interventions for psychosis and to compare the interventions across various clinical settings, i.e., inpatient, outpatient, rehabilitative, and mixed settings.

## Methods

### Inclusion and exclusion criteria

To ensure that the meta-analysis was of high quality, it included only randomized, English-speaking peer-reviewed studies. The studies had to have been published in English.

The study population consisted of adults with psychosis (ICD-10 codes: F20.X, F23.X, and F25.X; if only DSM codes reported, they were converted to the respective ICD-10 code). The included diagnoses were schizophrenia and its subtypes; acute transient psychotic disorders; and schizoaffective disorders^38^. Drug-induced or other organic psychoses were excluded.

Interventions were defined as relaxation techniques used to reduce stress as part of the treatment for psychosis patients. These techniques included mindfulness-based interventions, yoga, progressive muscle relaxation, autogenic training, qigong, tai chi, biofeedback, and breathing techniques. These techniques were selected because they are easily accessible to patients and were pooled because they based on relaxation and physiological stress reduction. Moreover, pooling reflects clinical practice, where such techniques are commonly applied as a functional group and statistical power was increased.

Mindfulness-based interventions were only partially included, as the focus of this meta-analysis was on relaxation techniques with an explicit and primary aim of physiological stress reduction. Approaches such as Mindfulness-Based Cognitive Therapy or Acceptance and Commitment Therapy were excluded because they are predominantly cognitively driven and include relaxation as only one component among several therapeutic mechanisms.

The primary outcome was defined as a reduction of stress and of anxiety and general psychotic symptoms. Secondary outcomes were a reduction of aggression and positive and negative symptoms and relapse prevention. Given that individuals with psychotic disorders frequently experience persistent impairments in quality of life and subjective well-being, even in the presence of symptomatic remission, we included these outcomes as endpoints in our meta-analysis. Further relevant outcomes were hospitalization rate, and duration of hospitalization. The resulting population, intervention, control, and outcome (PICO) criteria are shown in Table 1.

**Table 1:** Population, intervention, control, and outcome criteria.

Population	Patients with psychosis (ICD-10 codes F20.X, F23.X, and F25.X)
Intervention	Mindfulness-based interventions such as yoga and body scanning, tai chi, qigong, autogenic training, progressive muscle relaxation, biofeedback, and breathing techniques
Control	Treatment as usual, placebo treatment (e.g., exercises), and no treatment (e.g., waiting list)
Outcome	1. Reduction of stress and of anxiety and general psychotic symptoms
	2. Reduction of aggression and positive and negative symptoms; relapse prevention
	3. Quality of life, current well-being, and hospitalization rate and duration

### Search strategy

The literature search was performed in five databases, i.e., PubMed, Scopus, Web of Science, PsycINFO, and Cochrane Library. Studies published from January 1, 1994, to the date of the search, i.e., December 16, 2022, were considered. The search string was adjusted for every database according to its specifications, but the architecture and weighting of the terms remained the same. The following search terms were used: psychosis OR psychotic OR schizophrenia OR “schizo”; “yoga”; mindfulness” OR “mindfulness-based stress reduction” OR “mbsr” OR “mindfulness intervention” OR “mind body intervention*”; progressive muscle relaxation; “tai chi”; “qigong”; “biofeedback”; “breathing technique*”; “relaxation”; clinical trial OR comparative study OR randomized controlled trial OR meta-analysis.

For the complete search strings, see Supplementary material.

### Study selection

After the literature search was performed, all results were reformatted into RIS files. Duplicates were removed with the literature management software Mendeley, then a two-stage screening was performed by two team members (NS and CK) independently to identify the studies that were eligible for inclusion in the meta-analysis. Discrepancies in the screening were discussed separately with a third team member (CT). Screening was performed with the free online tool CADIMA (https://www.cadima.info/), which was developed by the Julius Kühn Institute and the Collaboration for Environmental Evidence and enables parallel, independent screening for PICO criteria. CADIMA can be used even without programming knowledge and supports the screening process in a team with independent reviewers^39^.

In addition, the reference lists of the selected studies were reviewed manually to find relevant studies that were not found by the literature search. For more details on all screened studies see Supplementary material.

### Data extraction

An Excel spreadsheet was used to record the data extracted from the selected studies. The definition of the data to be extracted was guided by the recommendations given in the Cochrane Handbook^40^. Data extraction included the following items: title, author, date of publication, country of study, study design, number of participants, distribution of participants to the intervention and control groups, use of antipsychotics and other medications, number of drop-outs (in percentages, if applicable), diagnoses, setting, disease stage (i.e., first episode or chronic), age, sex distribution, intervention, information on ethical approval, duration of the sessions, session frequency, number of sessions, information on the leaders of the interventions, follow-ups, co-interventions, control conditions, measurement methods of the defined outcomes with the respective results, adverse effects of the interventions, and study conclusions. This process was conducted independently by two reviewers to ensure that all data were extracted as accurately as possible. After independent data extraction, discrepancies were clarified and possible mistakes were corrected in a subsequent reviewer meeting with a third author.

### Quality assessment

The Cochrane risk-of-bias tool RoB2 was used to assess the risk of bias of the selected studies. This tool assesses the following areas: randomization process, deviations from planned interventions, missing outcome data, measurement of outcome, and selection of reported outcome. The assessment is performed by evaluating signal questions related to each type of bias, and the end result is an overall assessment of the risk of bias for each study^41^. Two independent team members (NS, CK performed the assessment and discussed any discrepancies with a third team member (CT). We also performed a quality analysis with the Grading of Recommendations Assessment, Development, and Evaluation (GRADE) approach, as recommended by the Cochrane Handbook^42^. Each outcome included in the meta-analysis was assessed with regard to study design, risk of bias, inconsistency, immediacy, imprecision, and publication bias (Supplemental material 3).

### Statistical analysis

RevMan 5.4.1 was used to perform the statistical analysis and generate forest plots^43^.

All results of the included studies were continuous data. Therefore, the statistical analysis used the standardized mean difference (SMD) and the random-effect model. In accordance with the introduction to meta-analyses by Borenstein et al. (2021), if the same rating scale was used in the subgroup analyses and we could assume that there was no heterogeneity, we used the mean difference (MD) using a confidence interval of 95% and a significance level of *p* < 0.05.

The analysis used post-measurement comparisons because in randomized trials, such comparisons may estimate the same magnitude as a comparison of changes from the pre-intervention baseline^44^ and most of the selected studies did not report the standard deviation of the difference between baseline and post-intervention measurements. The effect size was defined in terms of SMD, as follows^42^: SMD of 0.2, small effect size; SMD of 0.5, moderate effect size; and SMD of 0.8, large effect size. To ensure that the statistical procedures were correct, statistical advice was obtained from the statistician at the LVR Klinikum Düsseldorf.

### Heterogeneity

ReviewManager 5.4 was used to estimate heterogeneity as the I^2^ value. The I^2^ values were interpreted as follows (Deeks et al., 2019): 0–40%, probably no relevant heterogeneity; 30–60%, moderate heterogeneity; 50–90%, substantial heterogeneity; and 75–100%, significant heterogeneity.

### Subgroup analyses

Subgroup analyses were performed for the patient setting, i.e., inpatient, outpatient, rehabilitative, and mixed settings (rehabilitative settings were settings that were different from inpatient and outpatient settings, and mixed settings included patients from inpatient and outpatient settings).

## Results

### Study selection

**Fig. 1:**
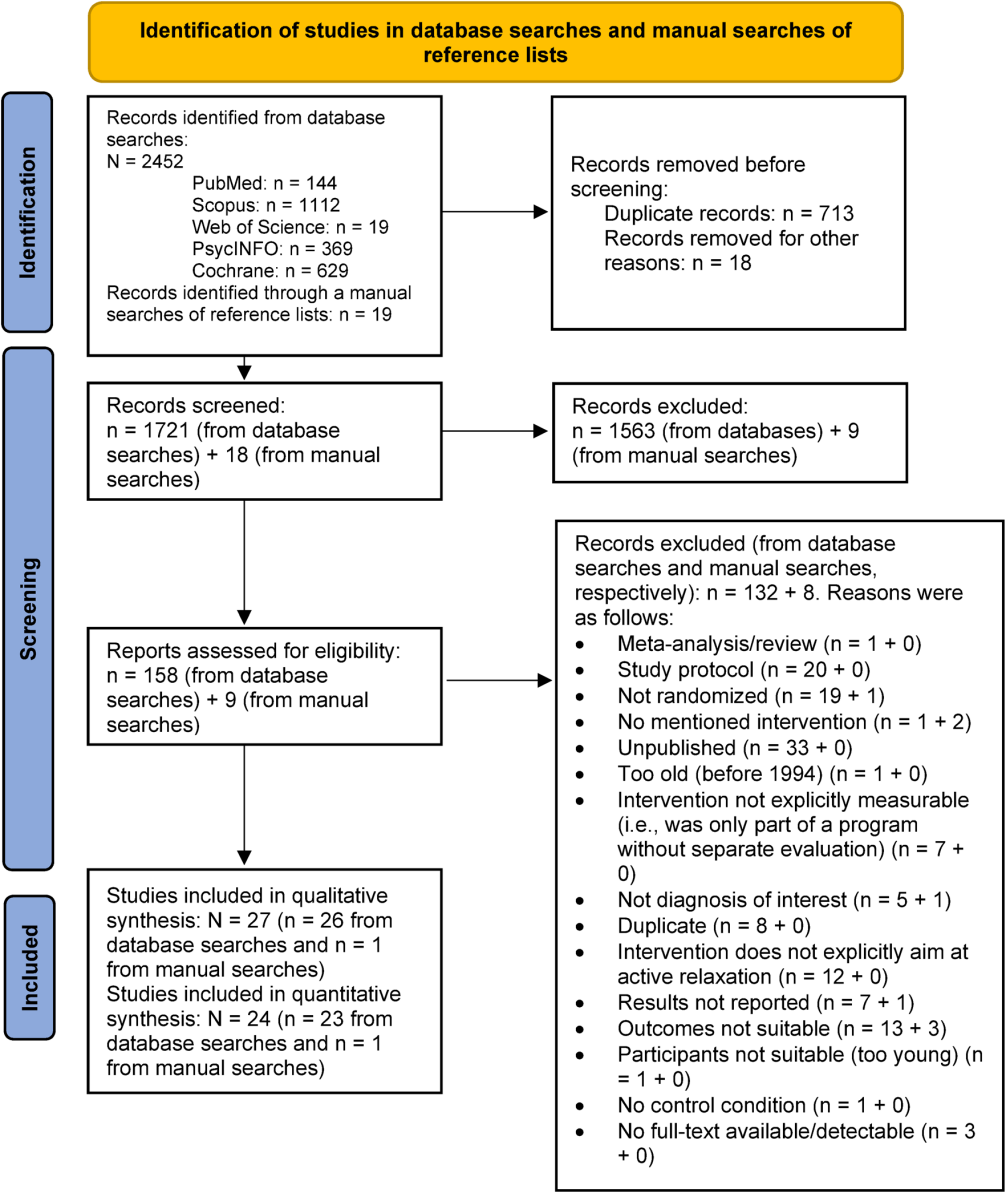
PRISMA flow diagram.

The search in the five databases yielded a total of 2452 hits. After the removal of duplicates and the screening process, 24 studies were eligible for inclusion. The details of the screening process are presented in a PRISMA flow diagram (Fig. 1).

After the initial search (performed on December 16, 2022), the search results were regularly updated throughout the analysis period, and any new articles that appeared to be suitable for the meta-analysis were reviewed. PubMed provided five new search results; Web of Science, 17; PsycINFO, 19; Cochrane library, nine; and Scopus, zero. However, none of the newly identified studies met the inclusion criteria, so none of them were included.

### Study characteristics

Altogether, 24 studies with a total of 1292 patients were included. The majority of the studies (*n* = 13) included a yoga intervention^45–57^. The other types of interventions were evaluation by the following number of studies: mindfulness-based training, four studies^58–61^; progressive muscle relaxation, three studies^62–64^; tai chi (in the form of 22 movement sequences from the Wu style in the Chen form of tai chi), two studies^65,66^; biofeedback (in the form of neurofeedback), one study^34^; and qigong (as a relaxation intervention), one study^30^.

The study settings were as follows: inpatient setting, seven studies^50,53,54,57,63,64,66^; outpatient setting, six studies^45,49,52,55,56,58^; rehabilitative setting, seven studies^30,34,46,60–62,65^; and mixed setting, four studies^47,48,51,59^. The patients were treated with an antipsychotic medication. For more information of the selected studies, see Supplemental 1.

### Quality assessment

The risks of bias calculated with the RoB2 were heterogeneous. None of the studies had a low risk of bias, 10 had a moderate risk^30,34,46,48,51,57,59,61,65,66^, and 14 had a high risk of bias^45,47,49,50,52–56,58,60,62–64^.

The assessment of quality by the GRADE approach indicated very low to low quality for the defined outcomes: The outcomes *general psychotic symptoms* and *quality of life* were of low quality, and the remaining outcomes were of very low quality. These results are mainly due to the heterogeneity between the studies and the risk of bias, which was high in many studies (cf. Supplemental material 3).

### Outcomes

Sensitivity analyses were conducted for studies showing high heterogeneity, and studies with a high risk of bias were excluded from both the overall and subgroup analyses. The results of the sensitivity analyses were consistent with the previously observed effects.

#### General meta-analysis

**Fig. 2:**
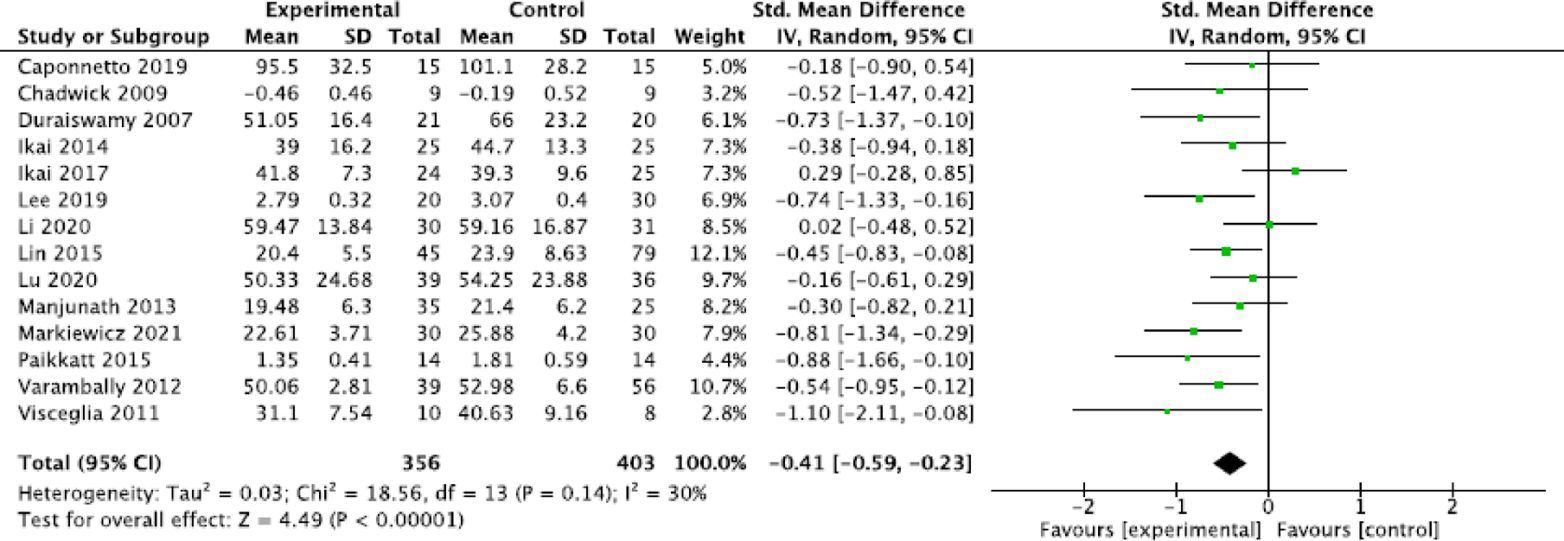
Forest plot general symptoms.

**Fig. 3:**
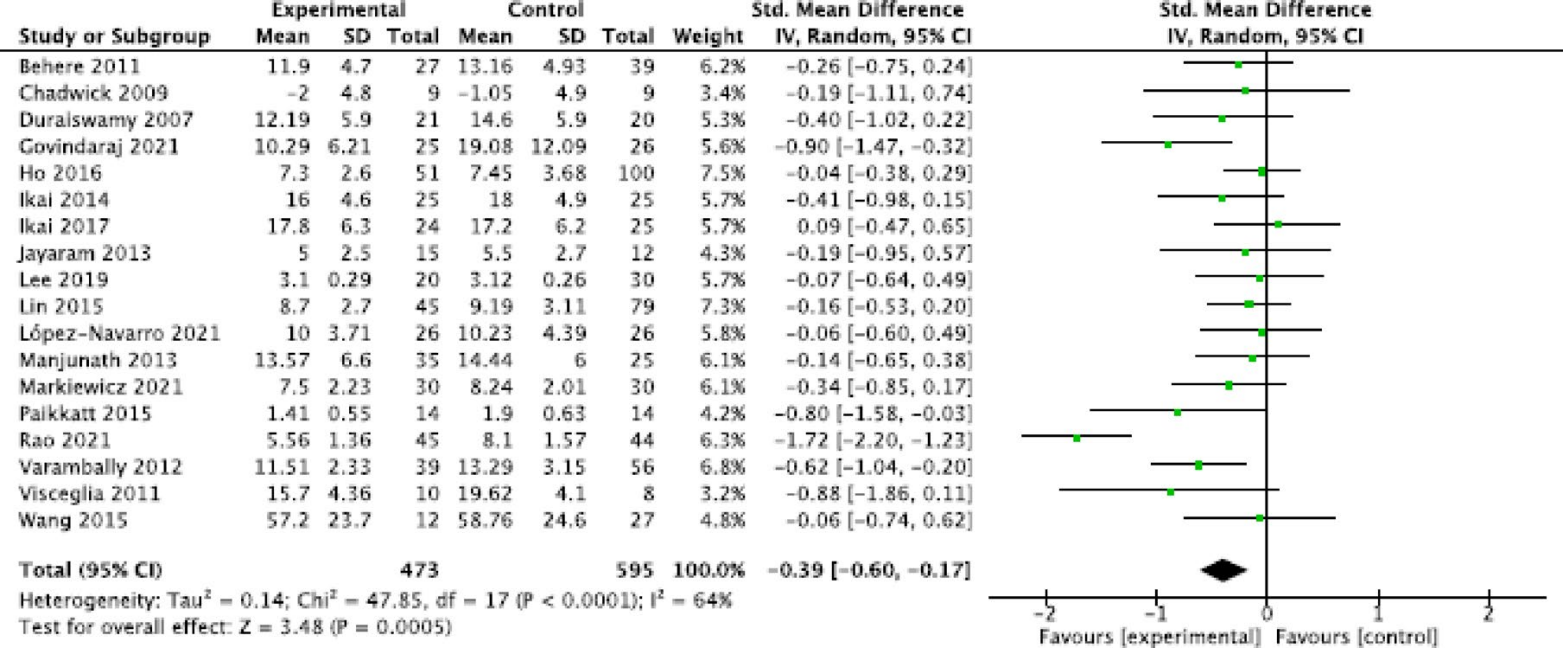
Forest plot positive symptoms.

**Fig. 4:**
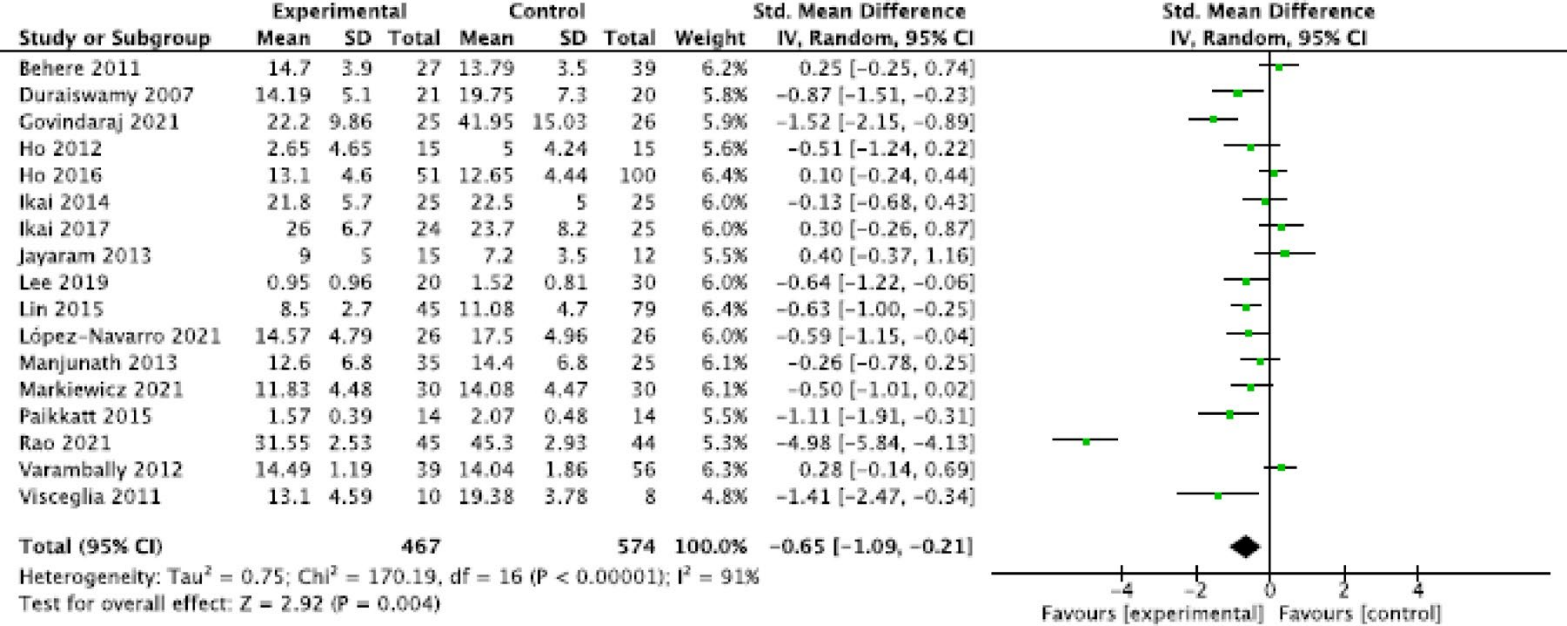
Forrest plot negative symptoms.

The general meta-analyses showed a significant moderate effect of relaxation techniques on the reduction of anxiety symptoms (three studies; SMD = −0.78, 95% CI = [−1.29, −0.27]; *P* = 0.002, I^2^ = 55%); a small, statistically significant positive effect on the reduction of general psychotic symptoms (14 studies; SMD = −0.41, 95% CI = [−0.59, −0.23], *P* < 0.00001, I^2^ = 30%; Fig. 2); a small, statistically significant positive effect on the reduction of positive symptoms (18 studies; SMD = −0.39, 95% CI = [−0.60, -0.17], *P* = 0.0005, I^2^ = 47%; Fig. 3); a moderate, statistically significant effect on the reduction of negative symptoms (17 studies; SMD = −0.65, 95% CI = [−1.09, −0.21], *P* = 0.004, I^2^ = 91%; Fig. 4); a moderate, statistically significant effect on improvement in quality of life (seven studies; SMD = −0.34, 95% CI = [−0.56, -0.12], *P* = 0.002, I^2^ = 5% ); a high, statistically significant effect on current well-being (one study with *N* = 52 patients; SMD = −1.25, 95% CI = [−1.84, −0.65], *P* < 0.0001) (Vancampfort et al., 011); and no significant effect for the reduction of stress (SMD = −0.27, 95% CI = [−1.06,0.52], *P* = 0.50, I^2^ = 85%).

None of the studies assessed actively adverse events. No adverse effects were reported in the studies.

#### Subgroup analysis

The subgroup analyses could be performed only for the outcomes of general psychotic symptoms, positive symptoms, negative symptoms, and quality of life and not for the other defined outcomes because for each of these outcomes, all the studies were performed in the same setting.

**Fig. 5:**
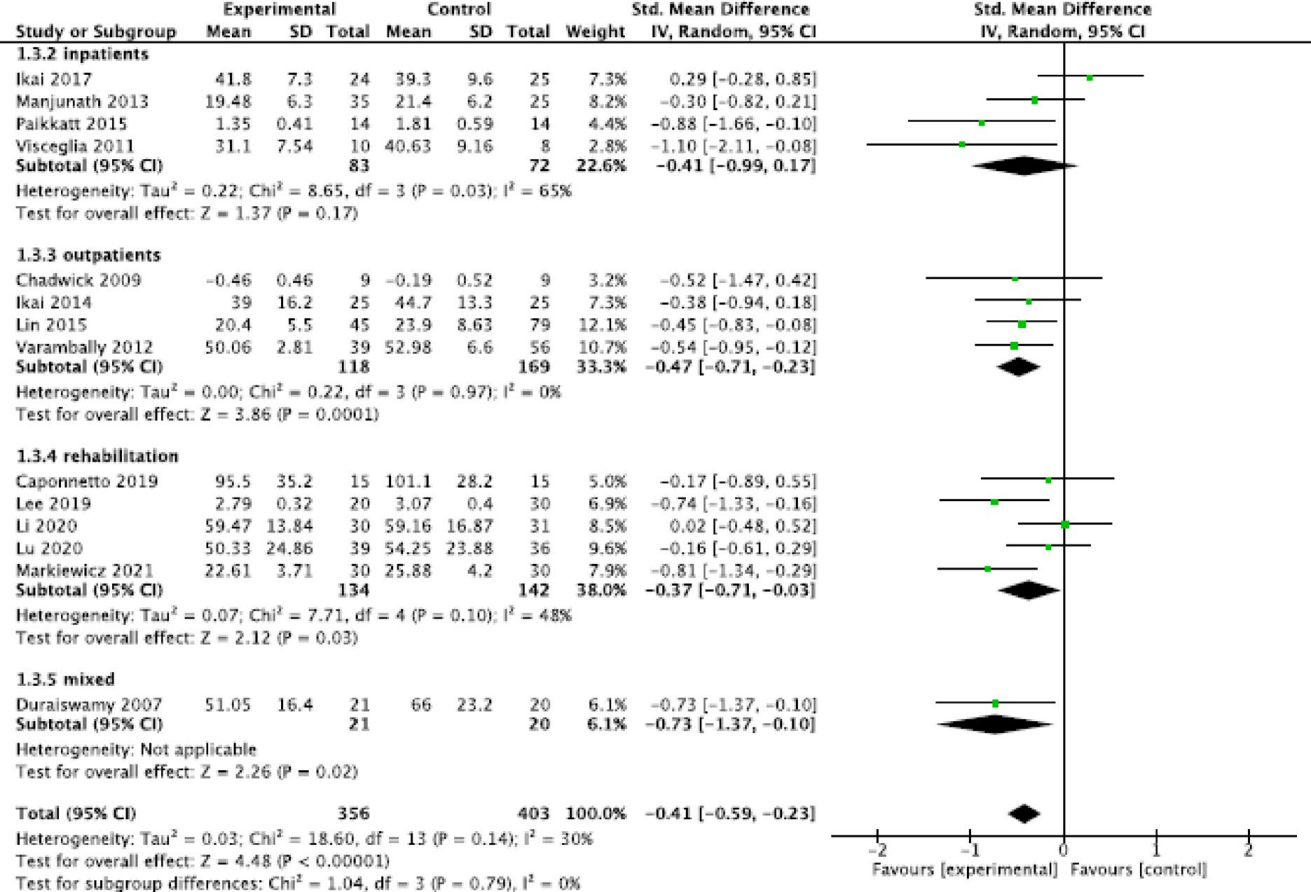
Forest plot general psychotic symptoms in all settings.

For the outcome general psychotic symptoms, the analysis showed a small significant effect in outpatient (SMD = −0.47, 95% CI = [−0.71, −0.23], *P* = 0.001, I^2^ = 0%) and rehabilitative settings (SMD = −0.37, 95% CI = [−0.71, −0.03], *P* = 0.03, I^2^ = 48%) but no significant effect in the inpatient setting (SMD = −0.41, 95% CI = [−0.99, 0.17], *P* = 0.17, I^2^ = 65%; Fig. 5). The one study that included patients in a mixed setting showed a moderate, statistically significant effect (SMD = −0.73, 95% CI = [−1.37, −0.10], *P* = 0.02).

For positive symptoms, the analysis found a moderate significant effect in outpatient (SMD = −0.58, 95% CI = [−1.07, −0.09], *P* = 0.02, I^2^ = 82%) and mixed settings (SMD = −0.54, 95% CI = [−0.95, −0.13], *P* = 0.01, I^2^ = 19%) but no significant effect in inpatient and rehabilitative settings (inpatient setting: SMD = −0.25, 95% CI = [−0.59, 0.10], *P* = 0.16, I^2^ = 26%; rehabilitative setting: SMD = −0.11, 95% CI = [−0.34, 0.12], *P* = 0.34, I^2^ = 0%).

The use of relaxation techniques did not show a statistically significant effect on the reduction of negative symptoms in any of the settings (inpatient: SMD = −0.50, 95% CI = [−1.06, 0.05], *P* = 0.08, I^2^ = 69%; outpatient: SMD = −0.99, 95% CI = [−2.25, 0.26], *P* = 0.12, I^2^ = 97%; rehabilitative: SMD = −0.36, 95% CI = [−0.77, 0.04], *P* = 0.08, I^2^ = 63%; mixed: SMD = −0.69, 95% CI = [−1.74, 0.37], *P* = 0.20, I^2^ = 86%)

The subgroup analysis regarding quality of life also showed no differences in the effects of relaxation techniques in inpatient, outpatient, and mixed settings (inpatient: SMD = −0.26, 95% CI = [−0.81, 0.29], *P* = 0.36, I^2^ = 0%; outpatient: SMD = −0.20, 95% CI = [−0.50, 0.11], *P* = 0.21, I^2^ = 0%; rehabilitative: SMD = −0.38, 95% CI = [−0.79, 0.04], *P* = 0.07, I^2^ = 8%). Only one study was performed in a mixed setting, but it showed a high, statistically significant effect (SMD = −0.95, 95% CI = [−1.60, −0.30], *P* = 0.004).

The sensitivity analyses performed for the respective outcomes in the subgroups in the presence of heterogeneity confirmed the above results (Supplemental material 2).

## Discussion

This meta-analysis on the use of relaxation techniques to reduce stress in psychosis patients included 24 studies with a total of 1292 patients. Most studies included only patients taking antipsychotics. Relaxation interventions had at least a small positive effect on all symptom areas, even if the effect was not always significant. The interventions had a moderate, significant effect in reducing anxiety symptoms and a significant effect on the reduction of general psychotic symptoms, as well as positive and negative symptoms. They also significantly improved patient quality of life. No adverse effects were reported. In general, the described effects could be confirmed by sensitivity analyses, which were performed in case of heterogeneity.

Several other meta-analyses have investigated the use of relaxation techniques in the treatment of psychotic patients^23–27,67,68^. However, none of them included subgroup analyses of various treatment settings for psychosis patients.

The finding that relaxation techniques seem not to have any adverse effects and did not lead to a symptom worsening has already been shown by various meta-analyses^23,26,67^. In line with this, our meta-analysis also suggest that the included relaxation techniques, i.e., yoga, mindfulness-based training, progressive muscle relaxation, tai chi, biofeedback, and qigong, neither showed a worsening of symptoms nor adverse events and it can be concluded that the interventions are safe for use in psychosis patients, especially in those taking medication. Nevertheless, the studies did not systematically and actively assess adverse events and did not report on possible intervention-related reasons for drop out or medication change, which may lead to an underreporting of potential (mild) adverse event. However, the absence of severe adverse effects legitimizes considering the use of the included relaxation methods as an add-on to medication in the treatment of psychosis patients and does not confirm previous findings in individual cases that the use of relaxation methods can trigger psychoses^18^.

The general meta-analysis found positive effects for the respective outcomes and showed no major differences between the interventions, even though stronger evidence is available for yoga interventions. Based on this result, we can conclude that all the included relaxation techniques can be considered to be used in all types of treatment settings (i.e., inpatient, outpatient, rehabilitative, and mixed) and certain settings may not require a specific program or procedure. Concluded from our subgroup analysis, outpatients and patients in a mixed setting benefitted most from the investigated relaxation techniques. This finding draws the attention to an appropriate time for the investigated interventions. Although the relaxation techniques do not harm when applied in the phase of an acute exacerbation of the psychotic disorder in an inpatient setting and may lead to an improvement in the perception of the inpatient stay, the effect seems to be more substantive in the post-acute phase. One reason for this may be the focus on finding the right medication in the right dosage in the inpatient settings, while patients in outpatient settings with a certain degree of symptom stability may have more mental capacity for the exercises. Positive effects in the mixed setting even for inpatients, may include motivational aspects when paired with more stable patients.

In conclusion, our results indicate that the choice of intervention may not be important, nevertheless more research is needed especially for tai chi, qui gong and biofeedback, as they there are limited numbers of studies compared to the other interventions. This finding may lead to a destigmatisation of psychosis patients because it means that they can participate in all types of existing relaxation approaches and are not excluded by their illness. This is an important finding in view of the increasing availability of relaxation approaches in society. Yoga courses in particular are widely offered nowadays, and demand is high. Therefore, it makes sense to establish yoga or similar interventions in clinical settings. Furthermore, our findings support the inclusion of the studied relaxation techniques in psychosis therapy in (inter)national guidelines.

Because relaxation interventions for stress reduction do not have to be specifically adapted to patients and patients can attend courses in their immediate environment, these interventions can be easily integrated into general treatment, which would increase patient self-efficacy. Higher self-efficacy may improve compliance because patients are more independent of their therapists and can actively shape their own therapy. Furthermore, it may also lead to an increased sense of well-being, which could also contribute to the success of the treatment. This point is particularly important when considering the poor recovery rate for psychosis patients^11^ because improving treatment success may also help to increase the recovery rate. Relaxation approaches could have additional positive effects on recovery by reducing psychotic symptoms and improving patients’ general state of health. Psychosis patients show a high prevalence of somatic comorbidities such as diabetes mellitus, cardiovascular disease, neurological disease, and pulmonary disease, which may be induced by the disease itself, a sedentary lifestyle, smoking, or adverse effects of psychotropic medication^6,7,9,69–71^. The high prevalence of such comorbidities is one reason why life expectancy is several years lower in psychosis patients^9,10^. Several studies have shown a positive effect of stress-reduction techniques on cardiovascular risk, body mass index, and the immune system^72–74^, so these approaches may reduce both the burden of the schizophrenia itself and the prevalence of somatic comorbidities and thus consequently reduce the economic burden of the disease^75^.

As described above, previous research indicates that the integration of relaxation techniques into treatment of psychotic disorders, especially schizophrenia may have advantages, and our meta-analysis provided further support for these advantages. Therefore, we suggest that guidelines for the treatment of psychotic disorders such as schizophrenia should consider to including relaxation techniques as a useful add-on treatment to standard therapy. However, it is striking that the meta-analysis, which focused on the reduction of stress through relaxation techniques, provided hardly any positive findings on the influence of these techniques, mainly because only three studies explicitly recorded stress reduction as an outcome and the studies otherwise focused primarily on symptoms. In any case, we can assume that stress and symptoms are closely linked, as proposed by the vulnerability-stress model^76^. Accordingly, it may not be necessary to analyze stress reduction directly as an outcome because a change in symptoms can also be used to draw conclusions about a change in stress. When the results of the meta-analysis are considered from this perspective, the positive effects of relaxation techniques could also be attributed to or linked to stress reduction, i.e., the results can be assumed to indirectly show a positive effect of relaxation techniques on the reduction of stress.

Across the included studies a high level of heterogeneity was observed. Several factors could have contributed to this finding. Firstly, the relaxation interventions differed in their theoretical background, structure, intensity and duration, ranging from movement-based approaches such as yoga and Tai Chi to more passive techniques such as progressive muscle relaxation or breathing exercises. Even within the intervention categories, there was considerable variation in implementation across the studies. Secondly, the included study populations showed heterogeneity regarding illness stage, symptom severity, and clinical setting. The most studies included chronically ill patients, while evidence for first-episode psychosis was limited. Moreover, psychiatric comorbidities could also contribute to heterogeneity as they are present in patients with psychotic disorders^5,69^ and may influence the effect of the intervention^31,77,78^. Additionally, outcome measures varied across the included studies, further contributing to between-study heterogeneity.

This heterogeneity limits the generalizability of pooled effect estimates and requires cautious interpretation of the results. Subgroup-analyses were conducted to explore potential sources of heterogeneity and suggested that yoga-based interventions were associated with more consistent effects. However, these findings should be interpreted carefully, as they were driven by a higher number of yoga studies compared to other relaxation techniques. Overall, the observed heterogeneity highlights the need for more standardized intervention protocols and outcome measures in future randomized controlled trials.

At the same time, the observed heterogeneity was part of the research aim, as this meta-analysis intentionally included a wide range of relaxation-based interventions and treatment settings to reflect real-world clinical practice. The persistence of beneficial effects across heterogeneous interventions and settings therefore supports the robustness and practical applicability of relaxation techniques.

While previous meta-analyses have demonstrated beneficial effects of relaxation-techniques for patients with psychotic disorders, they primarily focused on overall efficacy or the type of intervention. This meta-analysis extends the existing evidence by systematically examining the role of the treatment-setting through dedicated subgroup-analyses across inpatient, outpatient and rehabilitative contexts. To our knowledge, this is the first meta-analysis that specifically addresses the treatment-setting as a potential moderator of intervention effects. Additionally, this study focused on interventions with a clear and primary relaxation target. This approach reduced conceptual heterogeneity from cognitively driven techniques and allowed a more specific evaluation of relaxation-based interventions.

Several limitations should be acknowledged. The effect sizes were calculated using post-intervention values rather than change scores, as information required to estimate baseline-post correlations was not available across the included studies. While methodologically acceptable, this approach may be less sensitive to individual baseline differences. Furthermore, evidence for patients with first-episode psychosis was limited, which constrained the possibility of performing subgroup analyses by illness stage and therefore restricts the generalizability of the findings to early phases of the disorder. Finally, there was substantial heterogeneity in the applied relaxation interventions, with results largely driven by yoga-based studies, restricting conclusions regarding other relaxation techniques.

Conclusion: Relaxation techniques can be considered as add-on treatment approaches in various clinical settings in individuals with psychotic disorders when delivered as part of a physician-supervised treatment plan. Within such a structured medical framework, it may serve as a meaningful adjunctive intervention to support symptoms improvement and aid functional recovery. Relaxation techniques should be considered to be integrated into guidelines.

## Supplementary Information

Below is the link to the electronic supplementary material.

Supplementary Material 1

Supplementary Material 2

Supplementary Material 3

Supplementary Material 4

## Data Availability

The datasets used and/or analysed during the current study are available from the corresponding author on reasonable request.
