# Supplementary material for: A systematic review and meta-analysis of the impact of relaxation techniques to reduce burden of disease in patients with psychotic disorders
Source: Sci Rep. 2026 Mar 24;16:9841. doi: 10.1038/s41598-026-44310-0 (PMC13018224; doi:10.1038/s41598-026-44310-0)

Sensitivity analysis

- General Symptoms

➔ Subgroups analysis setting:

	Experimental			Control				Std. Mean Difference
**Study or Subgroup**	**Mean**	**SD**	**Total**	**Mean**	**SD**	**Total**	**Weight**	**IV, Random, 95% CI**
**1.3.2 inpatients**								
Ikai 2017	41.8	7.3	24	39.3	9.6	25	7.3%	0.29 [−0.28, 0.85]
Manjunath 2013	19.48	6.3	35	21.4	6.2	25	8.2%	−0.30 [−0.82, 0.21]
Paikkatt 2015	1.35	0.41	14	1.81	0.59	14	4.4%	−0.88 [−1.66, −0.10]
Visceglia 2011	31.1	7.54	10	40.63	9.16	8	2.8%	−1.10 [−2.11, −0.08]
**Subtotal (95% CI)**			**83**			**72**	**22.6%**	**−0.41 [−0.99, 0.17]**
Heterogeneity: $Tau^2 = 0.22; Chi^2 = 8.65, df = 3 (P = 0.03); I^2 = 65\%$								
Test for overall effect: Z = 1.37 (P = 0.17)								
**1.3.3 outpatients**								
Chadwick 2009	−0.46	0.46	9	−0.19	0.52	9	3.2%	−0.52 [−1.47, 0.42]
Ikai 2014	39	16.2	25	44.7	13.3	25	7.3%	−0.38 [−0.94, 0.18]
Lin 2015	20.4	5.5	45	23.9	8.63	79	12.1%	−0.45 [−0.83, −0.08]
Varambally 2012	50.06	2.81	39	52.98	6.6	56	10.7%	−0.54 [−0.95, −0.12]
**Subtotal (95% CI)**			**118**			**169**	**33.3%**	**−0.47 [−0.71, −0.23]**
Heterogeneity: $Tau^2 = 0.00; Chi^2 = 0.22, df = 3 (P = 0.97); I^2 = 0\%$								
Test for overall effect: Z = 3.86 (P = 0.0001)								
**1.3.4 rehabilitation**								
Caponnetto 2019	95.5	35.2	15	101.1	28.2	15	5.0%	−0.17 [−0.89, 0.55]
Lee 2019	2.79	0.32	20	3.07	0.4	30	6.9%	−0.74 [−1.33, −0.16]
Li 2020	59.47	13.84	30	59.16	16.87	31	8.5%	0.02 [−0.48, 0.52]
Lu 2020	50.33	24.86	39	54.25	23.88	36	9.6%	−0.16 [−0.61, 0.29]
Markiewicz 2021	22.61	3.71	30	25.88	4.2	30	7.9%	−0.81 [−1.34, −0.29]
**Subtotal (95% CI)**			**134**			**142**	**38.0%**	**−0.37 [−0.71, −0.03]**
Heterogeneity: $Tau^2 = 0.07; Chi^2 = 7.71, df = 4 (P = 0.10); I^2 = 48\%$								
Test for overall effect: Z = 2.12 (P = 0.03)								
**1.3.5 mixed**								
Duraiswamy 2007	51.05	16.4	21	66	23.2	20	6.1%	−0.73 [−1.37, −0.10]
**Subtotal (95% CI)**			**21**			**20**	**6.1%**	**−0.73 [−1.37, −0.10]**
Heterogeneity: Not applicable								
Test for overall effect: Z = 2.26 (P = 0.02)								
**Total (95% CI)**			**356**			**403**	**100.0%**	**−0.41 [−0.59, −0.23]**
Heterogeneity: $Tau^2 = 0.03; Chi^2 = 18.60, df = 13 (P = 0.14); I^2 = 30\%$								
Test for overall effect: Z = 4.48 (P < 0.00001)								
Test for subgroup differences: $Chi^2 = 1.04, df = 3 (P = 0.79), I^2 = 0\%$								

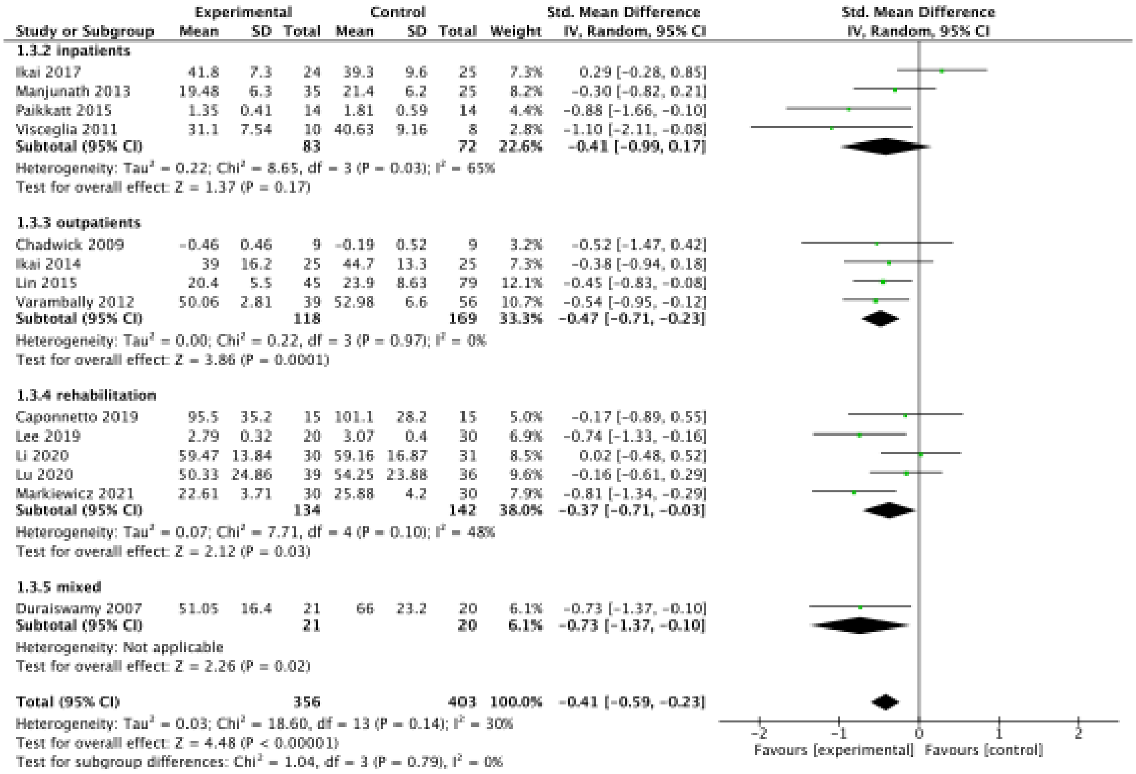

	Experimental			Control				Std. Mean Difference
**Study or Subgroup**	**Mean**	**SD**	**Total**	**Mean**	**SD**	**Total**	**Weight**	**IV, Random, 95% CI**
**1.3.2 inpatients**								
Ikai 2017	41.8	7.3	24	39.3	9.6	25	0.0%	0.29 [−0.28, 0.85]
Manjunath 2013	19.48	6.3	35	21.4	6.2	25	0.0%	−0.30 [−0.82, 0.21]
Paikkatt 2015	1.35	0.41	14	1.81	0.59	14	0.0%	−0.88 [−1.66, −0.10]
Visceglia 2011	31.1	7.54	10	40.63	9.16	8	3.6%	−1.10 [−2.11, −0.08]
**Subtotal (95% CI)**			**10**			**8**	**3.6%**	**−1.10 [−2.11, −0.08]**
Heterogeneity: Not applicable								
Test for overall effect: Z = 2.12 (P = 0.03)								
**1.3.3 outpatients**								
Chadwick 2009	−0.46	0.46	9	−0.19	0.52	9	4.2%	−0.52 [−1.47, 0.42]
Ikai 2014	39	16.2	25	44.7	13.3	25	11.9%	−0.38 [−0.94, 0.18]
Lin 2015	20.4	5.5	45	23.9	8.63	79	27.2%	−0.45 [−0.83, −0.08]
Varambally 2012	50.06	2.81	39	52.98	6.6	56	21.6%	−0.54 [−0.95, −0.12]
**Subtotal (95% CI)**			**118**			**169**	**65.0%**	**−0.47 [−0.71, −0.23]**
Heterogeneity: $Tau^2 = 0.00; Chi^2 = 0.22, df = 3 (P = 0.97); I^2 = 0\%$								
Test for overall effect: Z = 3.86 (P = 0.0001)								
**1.3.4 rehabilitation**								
Caponnetto 2019	95.5	35.2	15	101.1	28.2	15	7.3%	−0.17 [−0.89, 0.55]
Lee 2019	2.79	0.32	20	3.07	0.4	30	0.0%	−0.74 [−1.33, −0.16]
Li 2020	59.47	13.84	30	59.16	16.87	31	14.8%	0.02 [−0.48, 0.52]
Lu 2020	50.33	24.86	39	54.25	23.88	36	0.0%	−0.16 [−0.61, 0.29]
Markiewicz 2021	22.61	3.71	30	25.88	4.2	30	0.0%	−0.81 [−1.34, −0.29]
**Subtotal (95% CI)**			**45**			**46**	**22.1%**	**−0.04 [−0.45, 0.37]**
Heterogeneity: $Tau^2 = 0.00; Chi^2 = 0.18, df = 1 (P = 0.67); I^2 = 0\%$								
Test for overall effect: Z = 0.20 (P = 0.84)								
**1.3.5 mixed**								
Duraiswamy 2007	51.05	16.4	21	66	23.2	20	9.3%	−0.73 [−1.37, −0.10]
**Subtotal (95% CI)**			**21**			**20**	**9.3%**	**−0.73 [−1.37, −0.10]**
Heterogeneity: Not applicable								
Test for overall effect: Z = 2.26 (P = 0.02)								
**Total (95% CI)**			**194**			**243**	**100.0%**	**−0.42 [−0.62, −0.23]**
Heterogeneity: $Tau^2 = 0.00; Chi^2 = 6.46, df = 7 (P = 0.49); I^2 = 0\%$								
Test for overall effect: Z = 4.30 (P < 0.0001)								
Test for subgroup differences: $Chi^2 = 6.05, df = 3 (P = 0.11), I^2 = 50.4\%$								

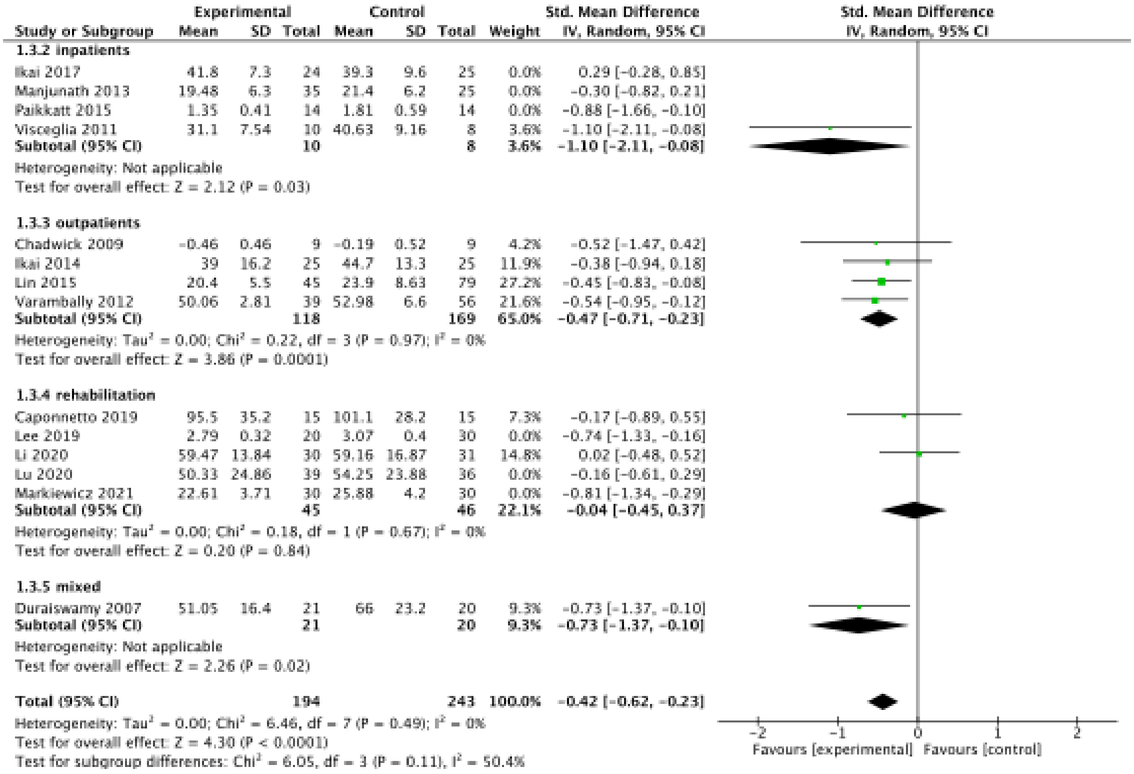

- Negative Symptoms

➔ General meta-analysis

Study or Subgroup	Experimental Mean	Experimental SD	Experimental Total	Control Mean	Control SD	Control Total	Weight	Std. Mean Difference IV, Random, 95% CI
Behere 2011	14.7	3.9	27	13.79	3.5	39	6.2%	0.25 [-0.25, 0.74]
Duraiswamy 2007	14.19	5.1	21	19.75	7.3	20	5.8%	-0.87 [-1.51, -0.23]
Govindaraj 2021	22.2	9.86	25	41.95	15.03	26	5.9%	-1.52 [-2.15, -0.89]
Ho 2012	2.65	4.65	15	5	4.24	15	5.6%	-0.51 [-1.24, 0.22]
Ho 2016	13.1	4.6	51	12.65	4.44	100	6.4%	0.10 [-0.24, 0.44]
Ikai 2014	21.8	5.7	25	22.5	5	25	6.0%	-0.13 [-0.68, 0.43]
Ikai 2017	26	6.7	24	23.7	8.2	25	6.0%	0.30 [-0.26, 0.87]
Jayaram 2013	9	5	15	7.2	3.5	12	5.5%	0.40 [-0.37, 1.16]
Lee 2019	0.95	0.96	20	1.52	0.81	30	6.0%	-0.64 [-1.22, -0.06]
Lin 2015	8.5	2.7	45	11.08	4.7	79	6.4%	-0.63 [-1.00, -0.25]
López-Navarro 2021	14.57	4.79	26	17.5	4.96	26	6.0%	-0.59 [-1.15, -0.04]
Manjunath 2013	12.6	6.8	35	14.4	6.8	25	6.1%	-0.26 [-0.78, 0.25]
Markiewicz 2021	11.83	4.48	30	14.08	4.47	30	6.1%	-0.50 [-1.01, 0.02]
Paikkatt 2015	1.57	0.39	14	2.07	0.48	14	5.5%	-1.11 [-1.91, -0.31]
Rao 2021	31.55	2.53	45	45.3	2.93	44	5.3%	-4.98 [-5.84, -4.13]
Varambally 2012	14.49	1.19	39	14.04	1.86	56	6.3%	0.28 [-0.14, 0.69]
Visceglia 2011	13.1	4.59	10	19.38	3.78	8	4.8%	-1.41 [-2.47, -0.34]
**Total (95% CI)**			**467**			**574**	**100.0%**	**-0.65 [-1.09, -0.21]**

Heterogeneity: $Tau^2 = 0.75$; $Chi^2 = 170.19$, df = 16 ($P < 0.00001$); $I^2 = 91\%$
Test for overall effect: Z = 2.92 (P = 0.004)

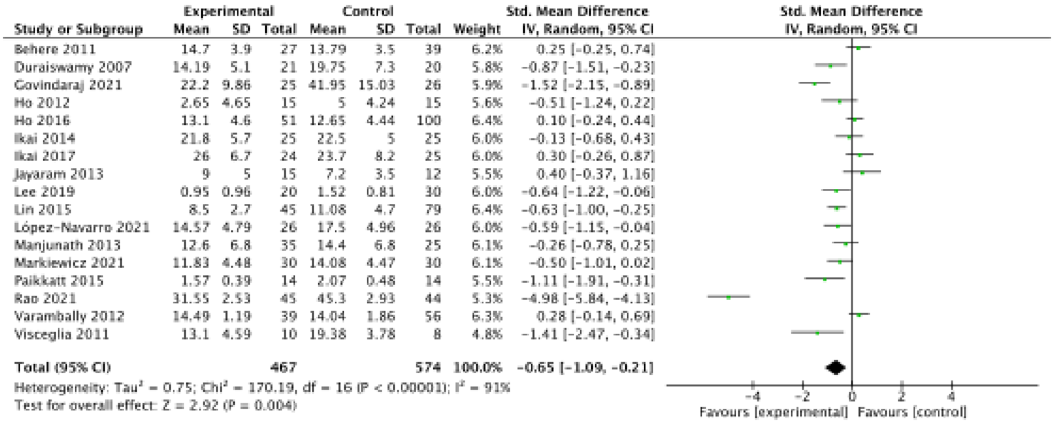

Study or Subgroup	Experimental Mean	Experimental SD	Experimental Total	Control Mean	Control SD	Control Total	Weight	Std. Mean Difference IV, Random, 95% CI
Behere 2011	14.7	3.9	27	13.79	3.5	39	0.0%	0.25 [-0.25, 0.74]
Duraiswamy 2007	14.19	5.1	21	19.75	7.3	20	0.0%	-0.87 [-1.51, -0.23]
Govindaraj 2021	22.2	9.86	25	41.95	15.03	26	14.6%	-1.52 [-2.15, -0.89]
Ho 2012	2.65	4.65	15	5	4.24	15	13.5%	-0.51 [-1.24, 0.22]
Ho 2016	13.1	4.6	51	12.65	4.44	100	17.5%	0.10 [-0.24, 0.44]
Ikai 2014	21.8	5.7	25	22.5	5	25	0.0%	-0.13 [-0.68, 0.43]
Ikai 2017	26	6.7	24	23.7	8.2	25	0.0%	0.30 [-0.26, 0.87]
Jayaram 2013	9	5	15	7.2	3.5	12	13.1%	0.40 [-0.37, 1.16]
Lee 2019	0.95	0.96	20	1.52	0.81	30	0.0%	-0.64 [-1.22, -0.06]
Lin 2015	8.5	2.7	45	11.08	4.7	79	0.0%	-0.63 [-1.00, -0.25]
López-Navarro 2021	14.57	4.79	26	17.5	4.96	26	15.4%	-0.59 [-1.15, -0.04]
Manjunath 2013	12.6	6.8	35	14.4	6.8	25	0.0%	-0.26 [-0.78, 0.25]
Markiewicz 2021	11.83	4.48	30	14.08	4.47	30	15.8%	-0.50 [-1.01, 0.02]
Paikkatt 2015	1.57	0.39	14	2.07	0.48	14	0.0%	-1.11 [-1.91, -0.31]
Rao 2021	31.55	2.53	45	45.3	2.93	44	0.0%	-4.98 [-5.84, -4.13]
Varambally 2012	14.49	1.19	39	14.04	1.86	56	0.0%	0.28 [-0.14, 0.69]
Visceglia 2011	13.1	4.59	10	19.38	3.78	8	10.1%	-1.41 [-2.47, -0.34]
**Total (95% CI)**			**172**			**217**	**100.0%**	**-0.53 [-1.03, -0.04]**

Heterogeneity: $Tau^2 = 0.34$; $Chi^2 = 28.80$, df = 6 ($P < 0.0001$); $I^2 = 79\%$
Test for overall effect: Z = 2.11 (P = 0.03)

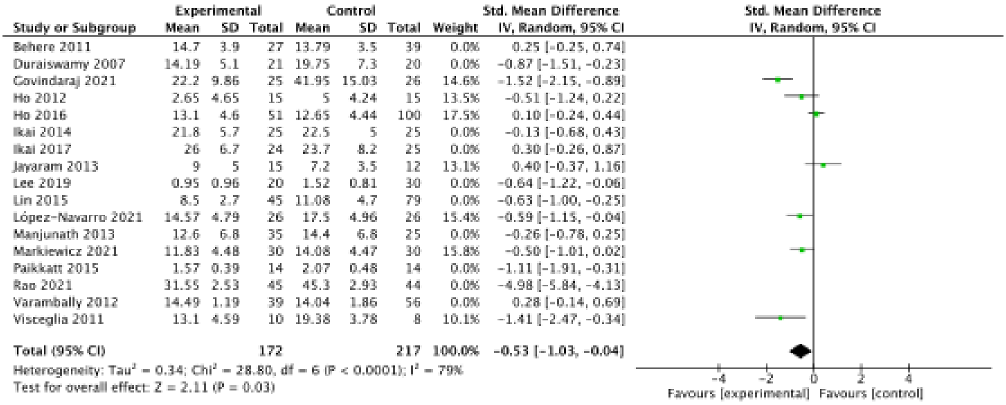

➔ Subgroup analysis interventions

	Experimental			Control				Std. Mean Difference
**Study or Subgroup**	**Mean**	**SD**	**Total**	**Mean**	**SD**	**Total**	**Weight**	**IV, Random, 95% CI**
**1.16.1 Yoga**								
Behere 2011	14.7	3.9	27	13.79	3.5	39	6.2%	0.25 [−0.25, 0.74]
Duraiswamy 2007	14.19	5.1	21	19.75	7.3	20	5.8%	−0.87 [−1.51, −0.23]
Govindaraj 2021	22	9.86	25	41.95	15.03	26	5.9%	−1.54 [−2.17, −0.91]
Ikai 2014	21.8	5.7	25	22.5	5	25	6.0%	−0.13 [−0.68, 0.43]
Ikai 2017	26	6.7	24	23.7	8.2	25	6.0%	0.30 [−0.26, 0.87]
Jayaram 2013	9	5	15	7.2	3.5	12	5.5%	0.40 [−0.37, 1.16]
Lin 2015	8.5	2.7	45	11.08	4.7	79	6.4%	−0.63 [−1.00, −0.25]
Manjunath 2013	12.6	6.8	35	14.4	6.8	25	6.1%	−0.26 [−0.78, 0.25]
Paikkatt 2015	1.57	0.39	14	2.07	0.48	14	5.5%	−1.11 [−1.91, −0.31]
Rao 2021	31.55	2.53	45	45.3	2.93	44	5.3%	−4.98 [−5.84, −4.13]
Varambally 2012	14.49	1.19	39	14.04	1.86	56	6.3%	0.28 [−0.14, 0.69]
Visceglia 2011	13.1	4.59	10	19.38	3.78	8	4.8%	−1.41 [−2.47, −0.34]
**Subtotal (95% CI)**			**325**			**373**	**69.8%**	**−0.77 [−1.41, −0.13]**
Heterogeneity: $Tau^2 = 1.16$; $Chi^2 = 160.19$, df = 11 ($P < 0.00001$); $I^2 = 93\%$								
Test for overall effect: Z = 2.37 (P = 0.02)								
**1.16.2 MBI**								
Lee 2019	0.95	0.96	20	1.52	0.81	30	6.0%	−0.64 [−1.22, −0.06]
López−Navarro 2021	14.57	4.79	26	17.5	4.96	26	6.0%	−0.59 [−1.15, −0.04]
**Subtotal (95% CI)**			**46**			**56**	**12.0%**	**−0.62 [−1.02, −0.21]**
Heterogeneity: $Tau^2 = 0.00$; $Chi^2 = 0.02$, df = 1 (P = 0.90); $I^2 = 0\%$								
Test for overall effect: Z = 3.01 (P = 0.003)								
**1.16.3 PMR**								
**Subtotal (95% CI)**			**0**			**0**		**Not estimable**
Heterogeneity: Not applicable								
Test for overall effect: Not applicable								
**1.16.4 Biofeedback**								
Markiewicz 2021	11.83	4.48	30	14.08	4.47	30	6.1%	−0.50 [−1.01, 0.02]
**Subtotal (95% CI)**			**30**			**30**	**6.1%**	**−0.50 [−1.01, 0.02]**
Heterogeneity: Not applicable								
Test for overall effect: Z = 1.89 (P = 0.06)								
**1.16.5 Tai chi**								
Ho 2012	2.65	4.65	15	5	4.24	15	5.6%	−0.51 [−1.24, 0.22]
Ho 2016	13.1	4.6	51	12.65	4.44	100	6.4%	0.10 [−0.24, 0.44]
**Subtotal (95% CI)**			**66**			**115**	**12.1%**	**−0.12 [−0.69, 0.46]**
Heterogeneity: $Tau^2 = 0.10$; $Chi^2 = 2.24$, df = 1 (P = 0.13); $I^2 = 55\%$								
Test for overall effect: Z = 0.40 (P = 0.69)								
**1.16.6 Qigong**								
**Subtotal (95% CI)**			**0**			**0**		**Not estimable**
Heterogeneity: Not applicable								
Test for overall effect: Not applicable								
**Total (95% CI)**			**467**			**574**	**100.0%**	**−0.65 [−1.09, −0.21]**
Heterogeneity: $Tau^2 = 0.75$; $Chi^2 = 170.47$, df = 16 ($P < 0.00001$); $I^2 = 91\%$								
Test for overall effect: Z = 2.92 (P = 0.004)								
Test for subgroup differences: $Chi^2 = 2.69$, df = 3 (P = 0.44), $I^2 = 0\%$								

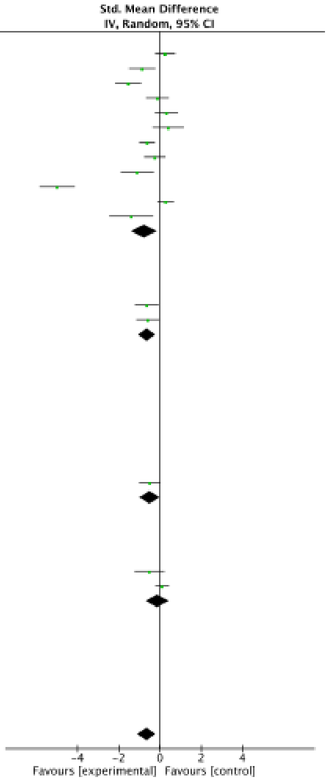

	Experimental			Control				Std. Mean Difference
**Study or Subgroup**	**Mean**	**SD**	**Total**	**Mean**	**SD**	**Total**	**Weight**	**IV, Random, 95% CI**
**1.16.1 Yoga**								
Behere 2011	14.7	3.9	27	13.79	3.5	39	0.0%	0.25 [−0.25, 0.74]
Duraiswamy 2007	14.19	5.1	21	19.75	7.3	20	0.0%	−0.87 [−1.51, −0.23]
Govindaraj 2021	22	9.86	25	41.95	15.03	26	14.6%	−1.54 [−2.17, −0.91]
Ikai 2014	21.8	5.7	25	22.5	5	25	0.0%	−0.13 [−0.68, 0.43]
Ikai 2017	26	6.7	24	23.7	8.2	25	0.0%	0.30 [−0.26, 0.87]
Jayaram 2013	9	5	15	7.2	3.5	12	13.1%	0.40 [−0.37, 1.16]
Lin 2015	8.5	2.7	45	11.08	4.7	79	0.0%	−0.63 [−1.00, −0.25]
Manjunath 2013	12.6	6.8	35	14.4	6.8	25	0.0%	−0.26 [−0.78, 0.25]
Paikkatt 2015	1.57	0.39	14	2.07	0.48	14	0.0%	−1.11 [−1.91, −0.31]
Rao 2021	31.55	2.53	45	45.3	2.93	44	0.0%	−4.98 [−5.84, −4.13]
Varambally 2012	14.49	1.19	39	14.04	1.86	56	0.0%	0.28 [−0.14, 0.69]
Visceglia 2011	13.1	4.59	10	19.38	3.78	8	10.2%	−1.41 [−2.47, −0.34]
**Subtotal (95% CI)**			**50**			**46**	**37.8%**	**−0.84 [−2.14, 0.46]**
Heterogeneity: $Tau^2 = 1.14$; $Chi^2 = 15.75$, df = 2 (P = 0.0004); $I^2 = 87\%$								
Test for overall effect: Z = 1.27 (P = 0.21)								
**1.16.2 MBI**								
Lee 2019	0.95	0.96	20	1.52	0.81	30	0.0%	−0.64 [−1.22, −0.06]
López−Navarro 2021	14.57	4.79	26	17.5	4.96	26	15.4%	−0.59 [−1.15, −0.04]
**Subtotal (95% CI)**			**26**			**26**	**15.4%**	**−0.59 [−1.15, −0.04]**
Heterogeneity: Not applicable								
Test for overall effect: Z = 2.09 (P = 0.04)								
**1.16.3 PMR**								
**Subtotal (95% CI)**			**0**			**0**		**Not estimable**
Heterogeneity: Not applicable								
Test for overall effect: Not applicable								
**1.16.4 Biofeedback**								
Markiewicz 2021	11.83	4.48	30	14.08	4.47	30	15.8%	−0.50 [−1.01, 0.02]
**Subtotal (95% CI)**			**30**			**30**	**15.8%**	**−0.50 [−1.01, 0.02]**
Heterogeneity: Not applicable								
Test for overall effect: Z = 1.89 (P = 0.06)								
**1.16.5 Tai chi**								
Ho 2012	2.65	4.65	15	5	4.24	15	13.5%	−0.51 [−1.24, 0.22]
Ho 2016	13.1	4.6	51	12.65	4.44	100	17.5%	0.10 [−0.24, 0.44]
**Subtotal (95% CI)**			**66**			**115**	**31.0%**	**−0.12 [−0.69, 0.46]**
Heterogeneity: $Tau^2 = 0.10$; $Chi^2 = 2.24$, df = 1 (P = 0.13); $I^2 = 55\%$								
Test for overall effect: Z = 0.40 (P = 0.69)								
**1.16.6 Qigong**								
**Subtotal (95% CI)**			**0**			**0**		**Not estimable**
Heterogeneity: Not applicable								
Test for overall effect: Not applicable								
**Total (95% CI)**			**172**			**217**	**100.0%**	**−0.54 [−1.03, −0.04]**
Heterogeneity: $Tau^2 = 0.34$; $Chi^2 = 29.08$, df = 6 ($P < 0.0001$); $I^2 = 79\%$								
Test for overall effect: Z = 2.11 (P = 0.03)								
Test for subgroup differences: $Chi^2 = 1.89$, df = 3 (P = 0.59), $I^2 = 0\%$								

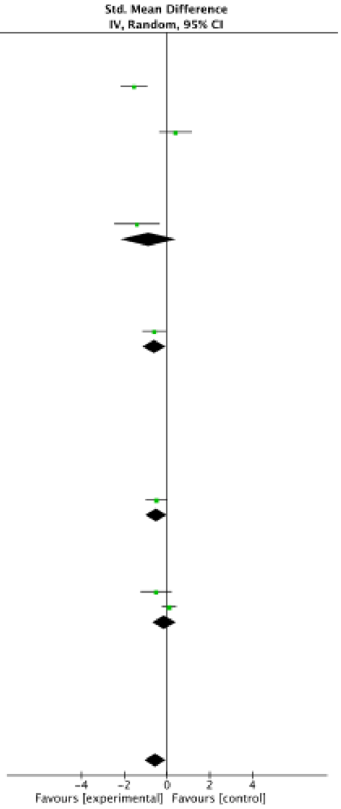

➔ Subgroup analysis setting

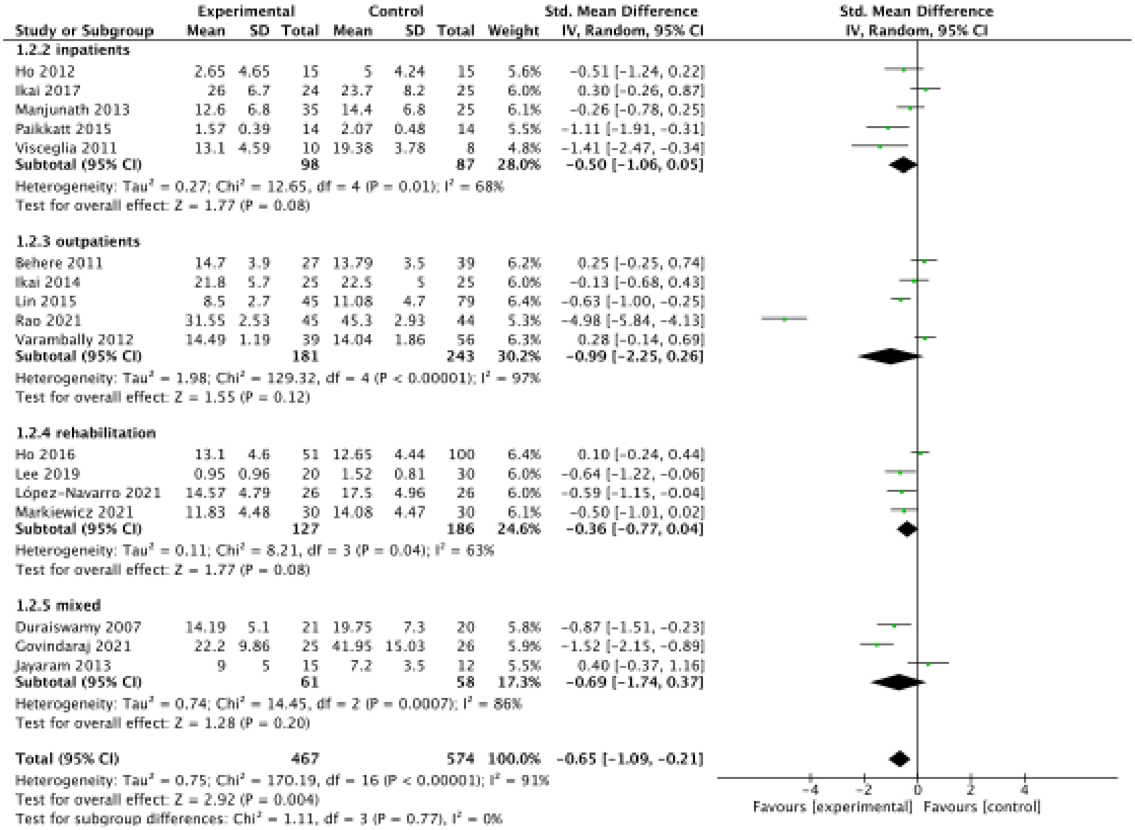

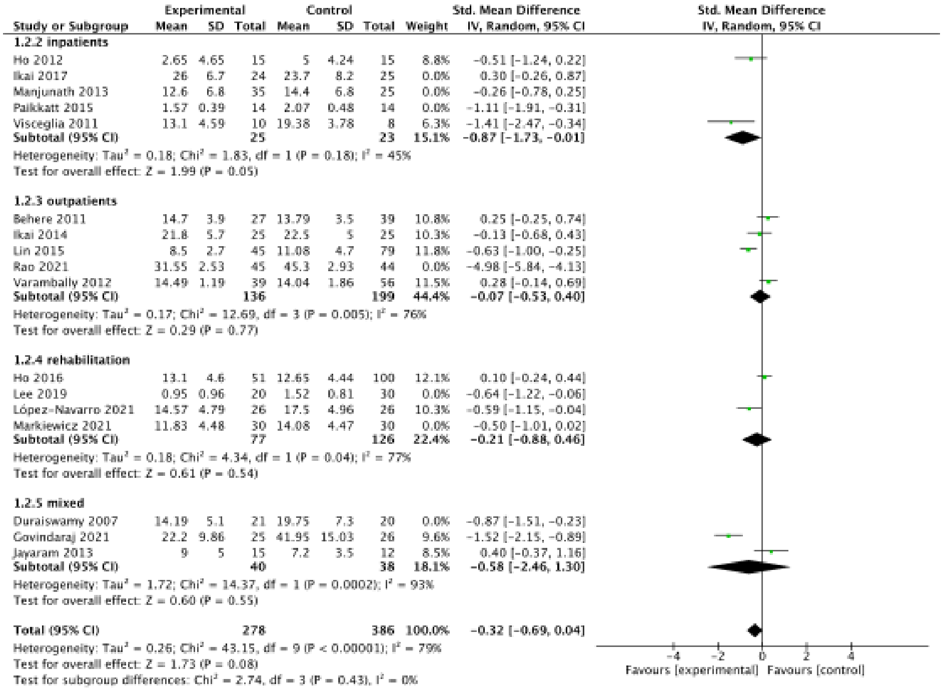

- Positive symptoms

➔ General meta-analysis

Study or Subgroup	Experimental Mean	Experimental SD	Experimental Total	Control Mean	Control SD	Control Total	Weight	Std. Mean Difference IV, Random, 95% CI
Behere 2011	11.9	4.7	27	13.16	4.93	39	6.2%	−0.26 [−0.75, 0.24]
Chadwick 2009	−2	4.8	9	−1.05	4.9	9	3.4%	−0.19 [−1.11, 0.74]
Duraiswamy 2007	12.19	5.9	21	14.6	5.9	20	5.3%	−0.40 [−1.02, 0.22]
Govindaraj 2021	10.29	6.21	25	19.08	12.09	26	5.6%	−0.90 [−1.47, −0.32]
Ho 2016	7.3	2.6	51	7.45	3.68	100	7.5%	−0.04 [−0.38, 0.29]
Ikai 2014	16	4.6	25	18	4.9	25	5.7%	−0.41 [−0.98, 0.15]
Ikai 2017	17.8	6.3	24	17.2	6.2	25	5.7%	0.09 [−0.47, 0.65]
Jayaram 2013	5	2.5	15	5.5	2.7	12	4.3%	−0.19 [−0.95, 0.57]
Lee 2019	3.1	0.29	20	3.12	0.26	30	5.7%	−0.07 [−0.64, 0.49]
Lin 2015	8.7	2.7	45	9.19	3.11	79	7.3%	−0.16 [−0.53, 0.20]
López-Navarro 2021	10	3.71	26	10.23	4.39	26	5.8%	−0.06 [−0.60, 0.49]
Manjunath 2013	13.57	6.6	35	14.44	6	25	6.1%	−0.14 [−0.65, 0.38]
Markiewicz 2021	7.5	2.23	30	8.24	2.01	30	6.1%	−0.34 [−0.85, 0.17]
Paikkatt 2015	1.41	0.55	14	1.9	0.63	14	4.2%	−0.80 [−1.58, −0.03]
Rao 2021	5.56	1.36	45	8.1	1.57	44	6.3%	−1.72 [−2.20, −1.23]
Varambally 2012	11.51	2.33	39	13.29	3.15	56	6.8%	−0.62 [−1.04, −0.20]
Visceglia 2011	15.7	4.36	10	19.62	4.1	8	3.2%	−0.88 [−1.86, 0.11]
Wang 2015	57.2	23.7	12	58.76	24.6	27	4.8%	−0.06 [−0.74, 0.62]
**Total (95% CI)**			**473**			**595**	**100.0%**	**−0.39 [−0.60, −0.17]**

Heterogeneity: $Tau^2 = 0.14$; $Chi^2 = 47.85$, df = 17 ($P < 0.0001$); $I^2 = 64\%$
Test for overall effect: Z = 3.48 (P = 0.0005)

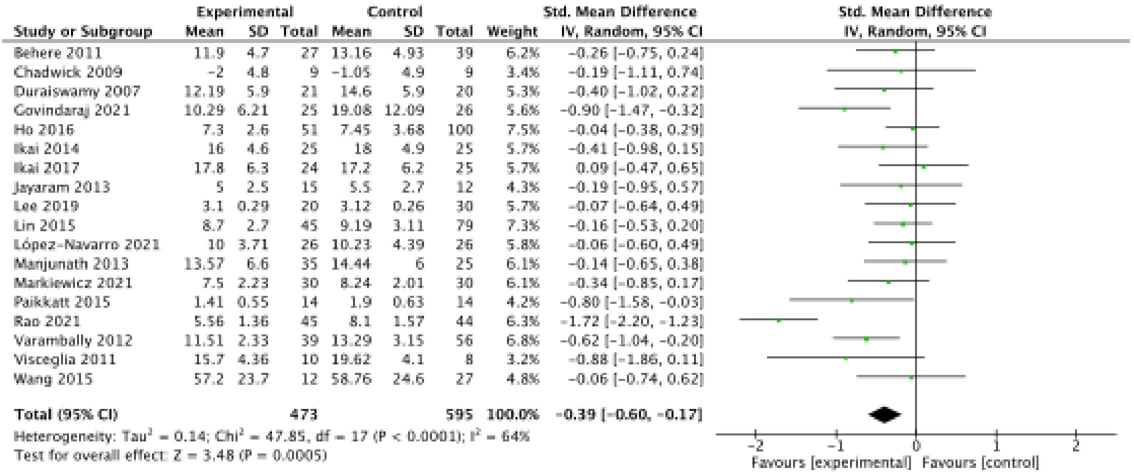

Study or Subgroup	Experimental Mean	Experimental SD	Experimental Total	Control Mean	Control SD	Control Total	Weight	Std. Mean Difference IV, Random, 95% CI
Behere 2011	11.9	4.7	27	13.16	4.93	39	16.6%	−0.26 [−0.75, 0.24]
Chadwick 2009	−2	4.8	9	−1.05	4.9	9	0.0%	−0.19 [−1.11, 0.74]
Duraiswamy 2007	12.19	5.9	21	14.6	5.9	20	0.0%	−0.40 [−1.02, 0.22]
Govindaraj 2021	10.29	6.21	25	19.08	12.09	26	13.2%	−0.90 [−1.47, −0.32]
Ho 2016	7.3	2.6	51	7.45	3.68	100	25.9%	−0.04 [−0.38, 0.29]
Ikai 2014	16	4.6	25	18	4.9	25	0.0%	−0.41 [−0.98, 0.15]
Ikai 2017	17.8	6.3	24	17.2	6.2	25	0.0%	0.09 [−0.47, 0.65]
Jayaram 2013	5	2.5	15	5.5	2.7	12	8.6%	−0.19 [−0.95, 0.57]
Lee 2019	3.1	0.29	20	3.12	0.26	30	0.0%	−0.07 [−0.64, 0.49]
Lin 2015	8.7	2.7	45	9.19	3.11	79	0.0%	−0.16 [−0.53, 0.20]
López-Navarro 2021	10	3.71	26	10.23	4.39	26	14.5%	−0.06 [−0.60, 0.49]
Manjunath 2013	13.57	6.6	35	14.44	6	25	0.0%	−0.14 [−0.65, 0.38]
Markiewicz 2021	7.5	2.23	30	8.24	2.01	30	15.8%	−0.34 [−0.85, 0.17]
Paikkatt 2015	1.41	0.55	14	1.9	0.63	14	0.0%	−0.80 [−1.58, −0.03]
Rao 2021	5.56	1.36	45	8.1	1.57	44	0.0%	−1.72 [−2.20, −1.23]
Varambally 2012	11.51	2.33	39	13.29	3.15	56	0.0%	−0.62 [−1.04, −0.20]
Visceglia 2011	15.7	4.36	10	19.62	4.1	8	5.5%	−0.88 [−1.86, 0.11]
Wang 2015	57.2	23.7	12	58.76	24.6	27	0.0%	−0.06 [−0.74, 0.62]
**Total (95% CI)**			**184**			**241**	**100.0%**	**−0.30 [−0.54, −0.06]**

Heterogeneity: $Tau^2 = 0.03$; $Chi^2 = 8.40$, df = 6 ($P = 0.21$); $I^2 = 29\%$
Test for overall effect: Z = 2.40 (P = 0.02)

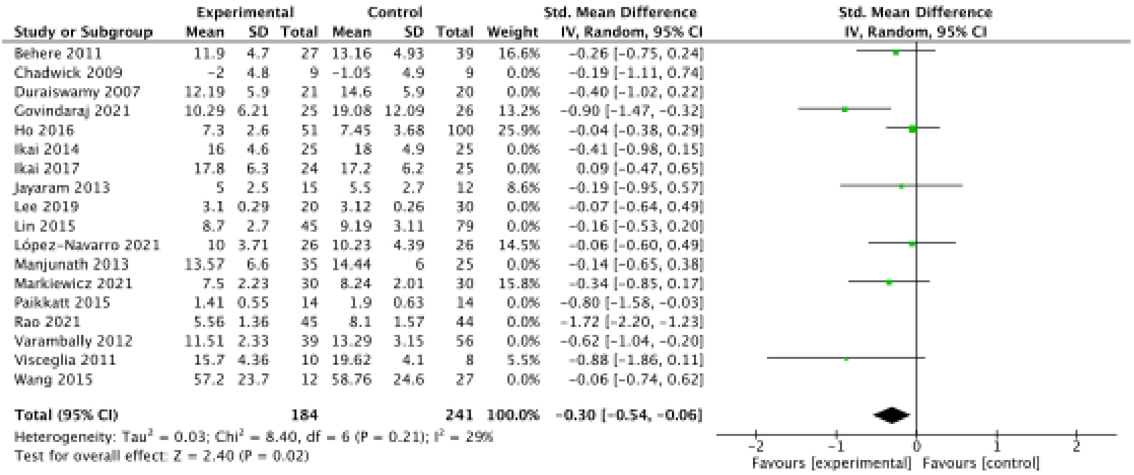

➔ Subgroup analysis intervention

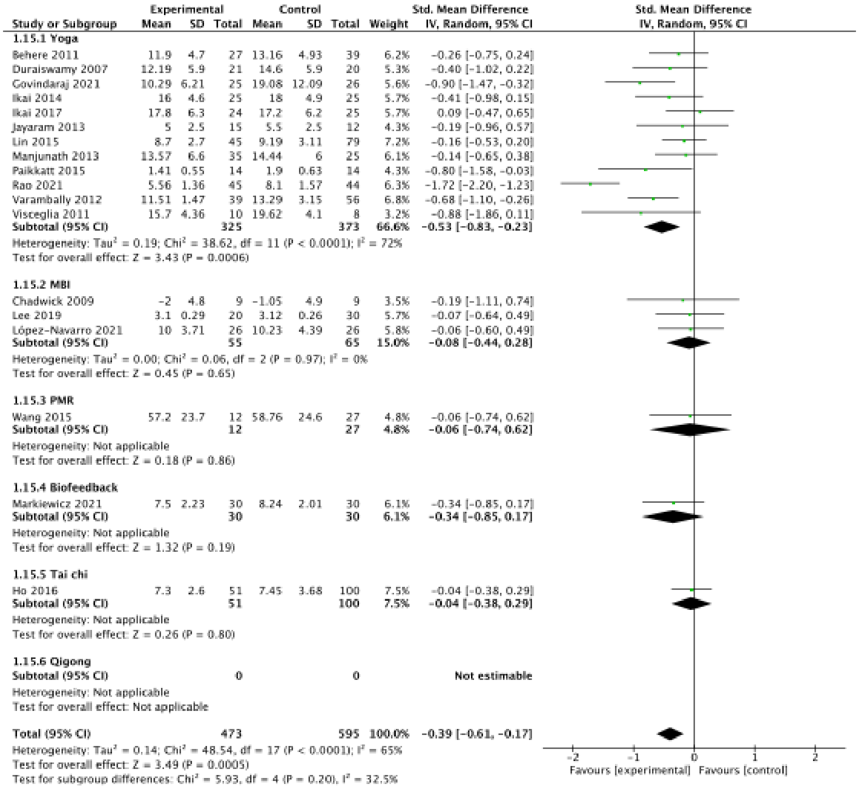

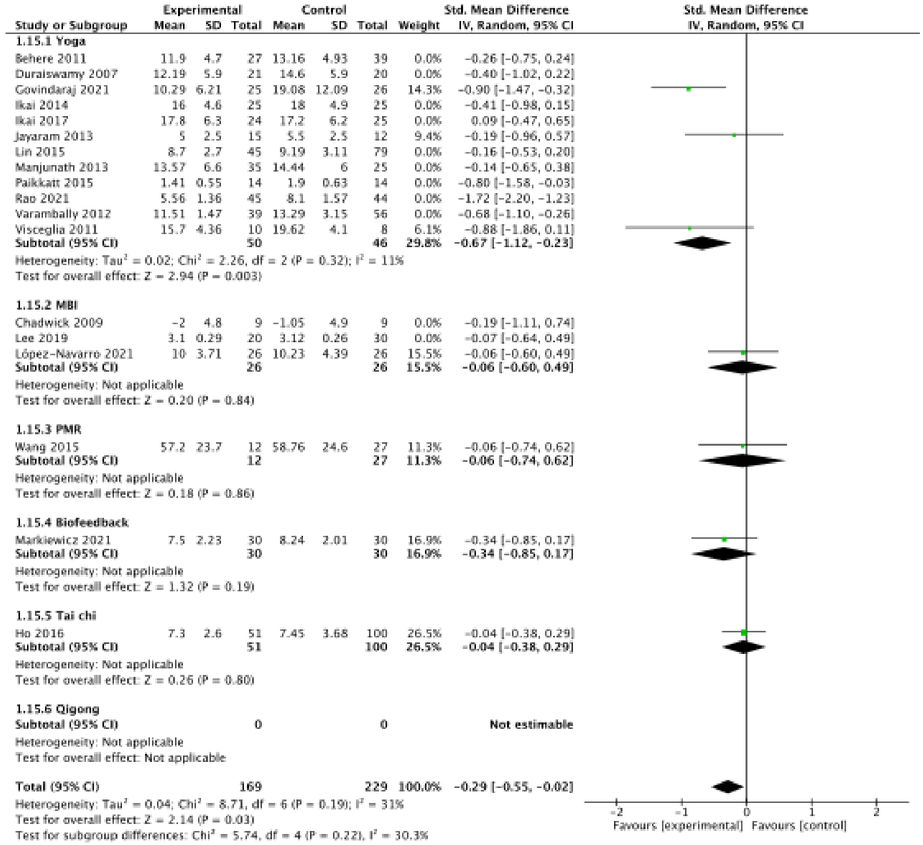

➔ Subgroup analysis setting

Study or Subgroup	Experimental Mean	Experimental SD	Experimental Total	Control Mean	Control SD	Control Total	Weight	Std. Mean Difference IV, Random, 95% CI
**1.1.2 inpatients**								
Ikai 2017	17.8	6.3	24	17.2	6.2	25	5.7%	0.09 [-0.47, 0.65]
Manjunath 2013	13.57	6.6	35	14.44	6	25	6.1%	-0.14 [-0.65, 0.38]
Paikkatt 2015	1.41	0.55	14	1.9	0.63	14	4.2%	-0.80 [-1.58, -0.03]
Visceglia 2011	15.7	4.36	10	19.62	4.1	8	3.2%	-0.88 [-1.86, 0.11]
Wang 2015	57.2	23.7	12	58.76	24.6	27	4.8%	-0.06 [-0.74, 0.62]
**Subtotal (95% CI)**			**95**			**99**	**24.0%**	**-0.25 [-0.59, 0.10]**
Heterogeneity: Tau² = 0.04; Chi² = 5.42, df = 4 (P = 0.25); I² = 26%								
Test for overall effect: Z = 1.40 (P = 0.16)								
**1.1.3 outpatients**								
Behere 2011	11.9	4.7	27	13.16	4.39	39	6.2%	-0.28 [-0.77, 0.22]
Chadwick 2009	-2	4.8	9	-1.05	4.9	9	3.4%	-0.19 [-1.11, 0.74]
Ikai 2014	16	4.6	25	18	4.9	25	5.7%	-0.41 [-0.98, 0.15]
Lin 2015	8.7	2.7	45	9.19	3.11	79	7.3%	-0.16 [-0.53, 0.20]
Rao 2021	5.56	1.36	45	8.1	1.57	44	6.3%	-1.72 [-2.20, -1.23]
Varambally 2012	11.51	2.33	39	13.29	3.15	56	6.8%	-0.62 [-1.04, -0.20]
**Subtotal (95% CI)**			**190**			**252**	**35.7%**	**-0.58 [-1.07, -0.09]**
Heterogeneity: Tau² = 0.29; Chi² = 28.18, df = 5 (P < 0.0001); I² = 82%								
Test for overall effect: Z = 2.33 (P = 0.02)								
**1.1.4 rehabilitation**								
Ho 2016	7.3	2.6	51	7.45	3.68	100	7.5%	-0.04 [-0.38, 0.29]
Lee 2019	3.1	0.29	20	3.12	0.26	30	5.7%	-0.07 [-0.64, 0.49]
López-Navarro 2021	10	3.71	26	10.23	4.39	26	5.8%	-0.06 [-0.60, 0.49]
Markiewicz 2021	7.5	2.23	30	8.24	2.01	30	6.1%	-0.34 [-0.85, 0.17]
**Subtotal (95% CI)**			**127**			**186**	**25.1%**	**-0.11 [-0.34, 0.12]**
Heterogeneity: Tau² = 0.00; Chi² = 1.01, df = 3 (P = 0.80); I² = 0%								
Test for overall effect: Z = 0.95 (P = 0.34)								
**1.1.5 mixed**								
Duraiswamy 2007	12.19	5.9	21	14.6	5.9	20	5.3%	-0.40 [-1.02, 0.22]
Govindaraj 2021	10.29	6.21	25	19.08	12.09	26	5.6%	-0.90 [-1.47, -0.32]
Jayaram 2013	5	2.5	15	5.5	2.7	12	4.3%	-0.19 [-0.95, 0.57]
**Subtotal (95% CI)**			**61**			**58**	**15.1%**	**-0.54 [-0.95, -0.13]**
Heterogeneity: Tau² = 0.03; Chi² = 2.47, df = 2 (P = 0.29); I² = 19%								
Test for overall effect: Z = 2.57 (P = 0.01)								
**Total (95% CI)**			**473**			**595**	**100.0%**	**-0.39 [-0.61, -0.17]**
Heterogeneity: Tau² = 0.13; Chi² = 47.79, df = 17 (P < 0.0001); I² = 64%								
Test for overall effect: Z = 3.49 (P = 0.0005)								
Test for subgroup differences: Chi² = 5.08, df = 3 (P = 0.17), I² = 41.0%								

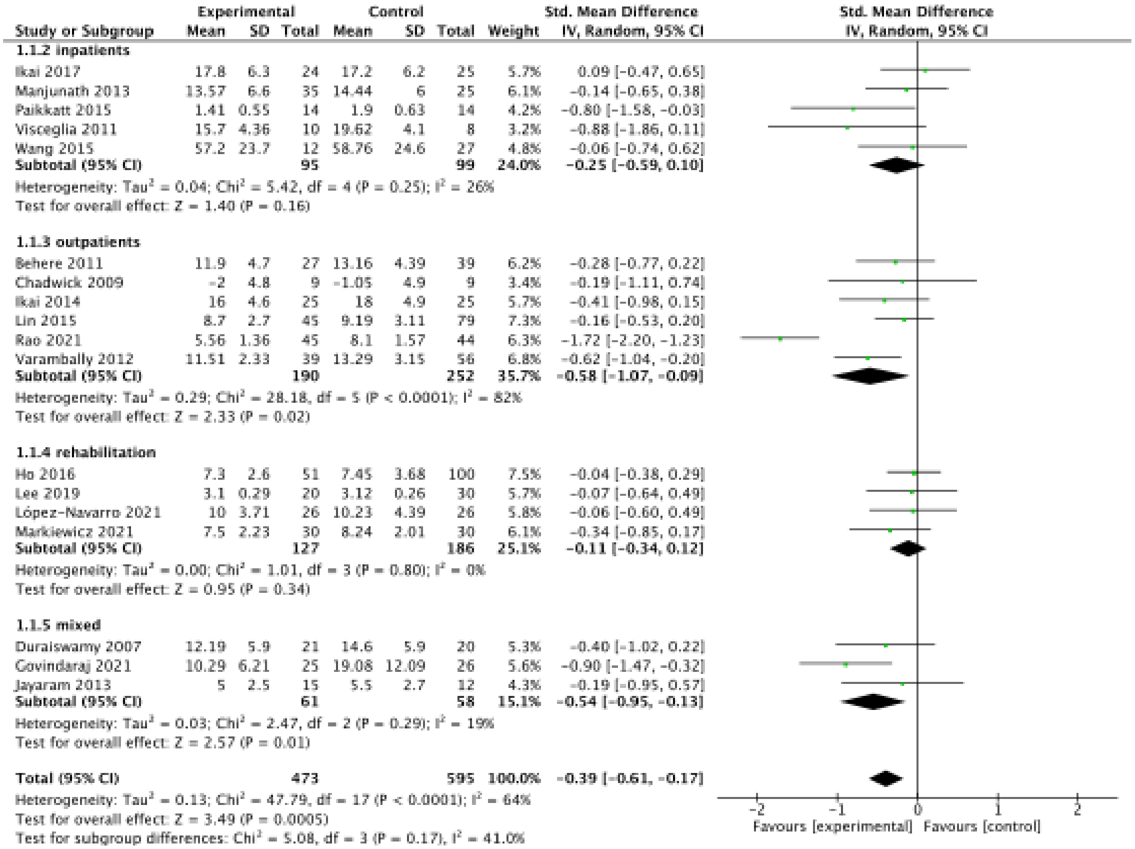

Study or Subgroup	Experimental Mean	Experimental SD	Experimental Total	Control Mean	Control SD	Control Total	Weight	Std. Mean Difference IV, Random, 95% CI
**1.1.2 inpatients**								
Ikai 2017	17.8	6.3	24	17.2	6.2	25	5.4%	0.09 [-0.47, 0.65]
Manjunath 2013	13.57	6.6	35	14.44	6	25	6.4%	-0.14 [-0.65, 0.38]
Paikkatt 2015	1.41	0.55	14	1.9	0.63	14	2.8%	-0.80 [-1.58, -0.03]
Visceglia 2011	15.7	4.36	10	19.62	4.1	8	1.8%	-0.88 [-1.86, 0.11]
Wang 2015	57.2	23.7	12	58.76	24.6	27	3.7%	-0.06 [-0.74, 0.62]
**Subtotal (95% CI)**			**95**			**99**	**20.0%**	**-0.25 [-0.59, 0.10]**
Heterogeneity: Tau² = 0.04; Chi² = 5.42, df = 4 (P = 0.25); I² = 26%								
Test for overall effect: Z = 1.40 (P = 0.16)								
**1.1.3 outpatients**								
Behere 2011	11.9	4.7	27	13.16	4.39	39	6.9%	-0.28 [-0.77, 0.22]
Chadwick 2009	-2	4.8	9	-1.05	4.9	9	2.0%	-0.19 [-1.11, 0.74]
Ikai 2014	16	4.6	25	18	4.9	25	5.4%	-0.41 [-0.98, 0.15]
Lin 2015	8.7	2.7	45	9.19	3.11	79	12.2%	-0.16 [-0.53, 0.20]
Rao 2021	5.56	1.36	45	8.1	1.57	44	0.0%	-1.72 [-2.20, -1.23]
Varambally 2012	11.51	2.33	39	13.29	3.15	56	9.5%	-0.62 [-1.04, -0.20]
**Subtotal (95% CI)**			**145**			**208**	**35.9%**	**-0.34 [-0.56, -0.13]**
Heterogeneity: Tau² = 0.00; Chi² = 2.85, df = 4 (P = 0.58); I² = 0%								
Test for overall effect: Z = 3.13 (P = 0.002)								
**1.1.4 rehabilitation**								
Ho 2016	7.3	2.6	51	7.45	3.68	100	14.2%	-0.04 [-0.38, 0.29]
Lee 2019	3.1	0.29	20	3.12	0.26	30	5.3%	-0.07 [-0.64, 0.49]
López-Navarro 2021	10	3.71	26	10.23	4.39	26	5.7%	-0.06 [-0.60, 0.49]
Markiewicz 2021	7.5	2.23	30	8.24	2.01	30	6.5%	-0.34 [-0.85, 0.17]
**Subtotal (95% CI)**			**127**			**186**	**31.7%**	**-0.11 [-0.34, 0.12]**
Heterogeneity: Tau² = 0.00; Chi² = 1.01, df = 3 (P = 0.80); I² = 0%								
Test for overall effect: Z = 0.95 (P = 0.34)								
**1.1.5 mixed**								
Duraiswamy 2007	12.19	5.9	21	14.6	5.9	20	4.4%	-0.40 [-1.02, 0.22]
Govindaraj 2021	10.29	6.21	25	19.08	12.09	26	5.1%	-0.90 [-1.47, -0.32]
Jayaram 2013	5	2.5	15	5.5	2.7	12	2.9%	-0.19 [-0.95, 0.57]
**Subtotal (95% CI)**			**61**			**58**	**12.4%**	**-0.54 [-0.95, -0.13]**
Heterogeneity: Tau² = 0.03; Chi² = 2.47, df = 2 (P = 0.29); I² = 19%								
Test for overall effect: Z = 2.57 (P = 0.01)								
**Total (95% CI)**			**428**			**551**	**100.0%**	**-0.27 [-0.40, -0.14]**
Heterogeneity: Tau² = 0.00; Chi² = 16.42, df = 16 (P = 0.42); I² = 3%								
Test for overall effect: Z = 4.05 (P < 0.0001)								
Test for subgroup differences: Chi² = 3.99, df = 3 (P = 0.26), I² = 24.8%								

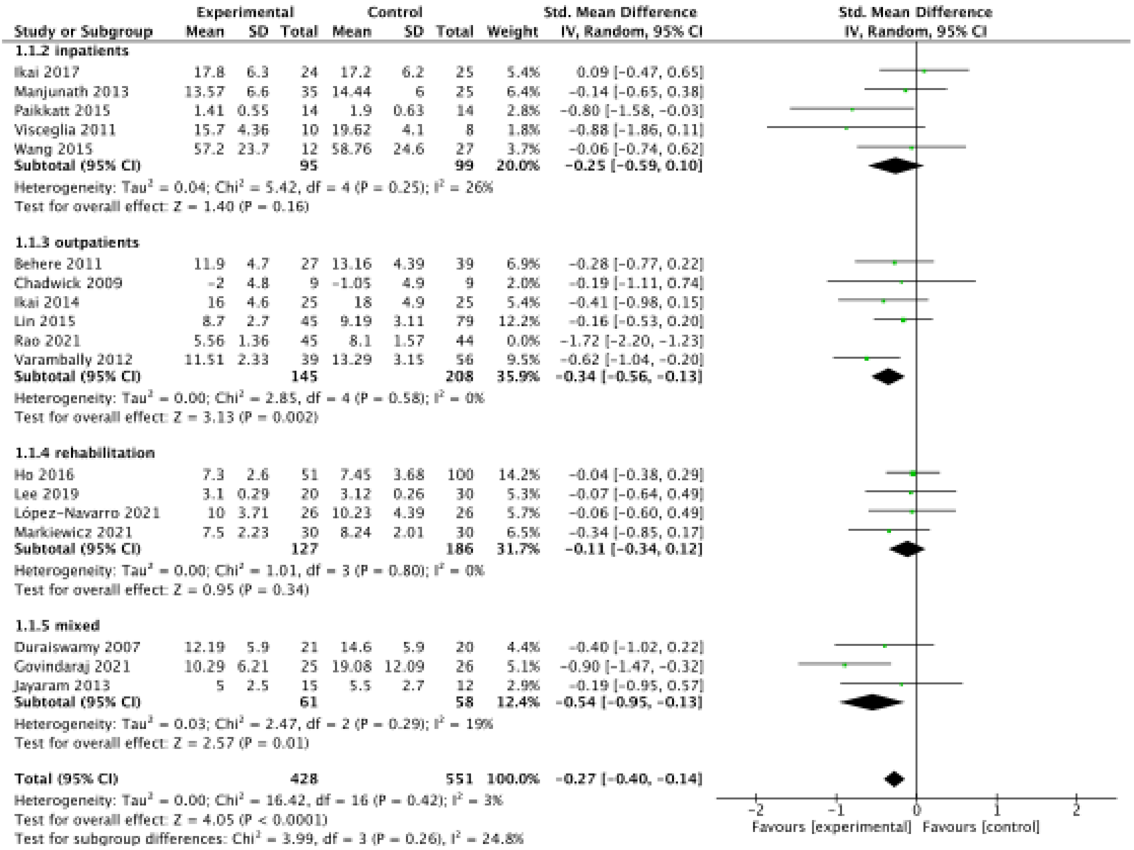

Supplement: Supplementary file 1 — Supplementary Material 1 [file 41598_2026_44310_MOESM1_ESM.pdf]
