# Supplementary material for: A systematic review and meta-analysis of the impact of relaxation techniques to reduce burden of disease in patients with psychotic disorders
Source: Sci Rep. 2026 Mar 24;16:9841. doi: 10.1038/s41598-026-44310-0 (PMC13018224; doi:10.1038/s41598-026-44310-0)
Supplement: Supplementary file 2 — Supplementary Material 2 [file 41598_2026_44310_MOESM2_ESM.pdf]

GRADE-Rating

Stress reduction: very low (-5)

GRADE criteria	Rating (circle one)	Footnotes (explain reasons for down- or upgrading)	Quality of the evidence (Circle one)
**Outcome:**			
**Study design**	RCT (starts as high quality)<br>Non-RCT (starts as low quality)	RCT = high	
**Risk of Bias**<br>*(use the Cochrane Risk of Bias tables and figures)*	No<br>serious (-1)<br>very serious (-2)	serious (2xsome concerns/1xhigh)= -1	⊕⊕⊕⊕<br>High
**Inconsistency**	No<br>serious (-1)<br>very serious (-2)	high heterogenity = -2	⊕⊕⊕◯<br>Moderate
**Indirectness**	No<br>serious (-1)<br>very serious (-2)	indirectness does not appear to be an issue = No	⊕⊕◯◯<br>Low
**Imprecision**	No<br>serious (-1)<br>very serious (-2)	less then 400/ lower confidence limit crosses SMD of 0.5 = -2	⊕◯◯◯<br>Very Low
**Publication Bias**	Undetected<br>Strongly suspected (-1)	not all results from small studies/ funnel plot not evaluable = 0	
**Other**<br>(upgrading factors, circle all that apply)	Large effect (+1 or +2)<br>Dose response (+1 or +2)<br>No Plausible confounding (+1 or +2)	Remember RCT evidence quality is very rarely upgraded.	

Anxiety reduction: very low (-5)

GRADE criteria	Rating (circle one)	Footnotes (explain reasons for down- or upgrading)	Quality of the evidence (Circle one)
**Outcome:**			
**Study design**	RCT (starts as high quality)<br>Non-RCT (starts as low quality)	RCT = high	
**Risk of Bias**<br>*(use the Cochrane Risk of Bias tables and figures)*	No<br>serious (-1)<br>very serious (-2)	very serious (1xsome concerns/2xhigh)= -2	⊕⊕⊕⊕<br>High
**Inconsistency**	No<br>serious (-1)<br>very serious (-2)	high heterogenity = -2	⊕⊕⊕◯<br>Moderate
**Indirectness**	No<br>serious (-1)<br>very serious (-2)	indirectness does not appear to be an issue = No	⊕⊕◯◯<br>Low
**Imprecision**	No<br>serious (-1)<br>very serious (-2)	less then 400/ upper and lower confidence limit crosses SMD of 0.5 = -2	⊕◯◯◯<br>Very Low
**Publication Bias**	Undetected<br>Strongly suspected (-1)	not all results from small studies/ funnel plot not evaluable = 0	
**Other**<br>(upgrading factors, circle all that apply)	Large effect (+1 or +2)<br>Dose response (+1 or +2)<br>No Plausible confounding (+1 or +2)	Remember RCT evidence quality is very rarely upgraded.	

Psychotic symptoms: low (-2)

GRADE criteria	Rating (circle one)	Footnotes (explain reasons for down- or upgrading)	Quality of the evidence (Circle one)
**Outcome:**			
**Study design**	RCT (starts as high quality)<br>Non-RCT (starts as low quality)	RCT = high	⊕⊕⊕⊕<br>High<br>⊕⊕⊕○<br>Moderate<br>⊕⊕○○<br>Low<br>⊕○○○<br>Very Low
**Risk of Bias**<br>*(use the Cochrane Risk of Bias tables and figures)*	No<br>serious (-1)<br>very serious (-2)	very serious (4xsome concerns/10xhigh)= -2	
**Inconsistency**	No<br>serious (-1)<br>very serious (-2)	low heterogenity = No	
**Indirectness**	No<br>serious (-1)<br>very serious (-2)	indirectness does not appear to be an issue = No	
**Imprecision**	No<br>serious (-1)<br>very serious (-2)	more than 400/ upper and lower confidence limit don´t cross SMD of 0.5 = No	
**Publication Bias**	Undetected<br>Strongly suspected (-1)	not all results from small studies/ funnel plot symmetrical = 0	
**Other**<br>(upgrading factors, circle all that apply)	Large effect (+1 or +2)<br>Dose response (+1 or +2)<br>No Plausible confounding (+1 or +2)	Remember RCT evidence quality is very rarely upgraded.	

Positive symptoms: very low (-3)

GRADE criteria	Rating (circle one)	Footnotes (explain reasons for down- or upgrading)	Quality of the evidence (Circle one)
**Outcome:**			
**Study design**	RCT (starts as high quality)<br>Non-RCT (starts as low quality)	RCT = high	⊕⊕⊕⊕<br>High<br>⊕⊕⊕○<br>Moderate<br>⊕⊕○○<br>Low<br>⊕○○○<br>Very Low
**Risk of Bias**<br>*(use the Cochrane Risk of Bias tables and figures)*	No<br>serious (-1)<br>very serious (-2)	very serious (6xsome concerns/12xhigh)= -2	
**Inconsistency**	No<br>serious (-1)<br>very serious (-2)	substantial heterogenity (64%) = -1	
**Indirectness**	No<br>serious (-1)<br>very serious (-2)	indirectness does not appear to be an issue = No	
**Imprecision**	No<br>serious (-1)<br>very serious (-2)	more than 400/ upper and lower confidence limit don´t cross SMD of 0.5 = No	
**Publication Bias**	Undetected<br>Strongly suspected (-1)	not all results from small studies/ funnel plot seems to be symmetrical = 0	
**Other**<br>(upgrading factors, circle all that apply)	Large effect (+1 or +2)<br>Dose response (+1 or +2)<br>No Plausible confounding (+1 or +2)	Remember RCT evidence quality is very rarely upgraded.	

Negative symptoms: very low (-4)

GRADE criteria	Rating (circle one)	Footnotes (explain reasons for down- or upgrading)	Quality of the evidence (Circle one)
**Outcome:**			
**Study design**	RCT (starts as high quality)<br>Non-RCT (starts as low quality)	RCT = high	⊕⊕⊕⊕ High<br>⊕⊕⊕○ Moderate<br>⊕⊕○○ Low<br>⊕○○○ Very Low
**Risk of Bias** *(use the Cochrane Risk of Bias tables and figures)*	No<br>serious (-1)<br>very serious (-2)	very serious (7xsome concerns/10xhigh)= -2	
**Inconsistency**	No<br>serious (-1)<br>very serious (-2)	high heterogenity (91%) = -2	
**Indirectness**	No<br>serious (-1)<br>very serious (-2)	indirectness does not appear to be an issue = No	
**Imprecision**	No<br>serious (-1)<br>very serious (-2)	more than 400/ upper and lower confidence limit cross SMD of 0.5 = No	
**Publication Bias**	Undetected<br>Strongly suspected (-1)	not all results from small studies/ funnel plot seems to be symmetrical = 0	
**Other** (upgrading factors, circle all that apply)	Large effect (+1 or +2)<br>Dose response (+1 or +2)<br>No Plausible confounding (+1 or +2)	Remember RCT evidence quality is very rarely upgraded.	

Well-being: very low (-4)

GRADE criteria	Rating (circle one)	Footnotes (explain reasons for down- or upgrading)	Quality of the evidence (Circle one)
**Outcome:**			
**Study design**	RCT (starts as high quality)<br>Non-RCT (starts as low quality)	RCT = high	⊕⊕⊕⊕ High<br>⊕⊕⊕○ Moderate<br>⊕⊕○○ Low<br>⊕○○○ Very Low
**Risk of Bias** *(use the Cochrane Risk of Bias tables and figures)*	No<br>serious (-1)<br>very serious (-2)	very serious (1xhigh)= -2	
**Inconsistency**	No<br>serious (-1)<br>very serious (-2)	only one study = No	
**Indirectness**	No<br>serious (-1)<br>very serious (-2)	indirectness does not appear to be an issue = No	
**Imprecision**	No<br>serious (-1)<br>very serious (-2)	less than 400/ upper and lower confidence limit cross SMD of 0.5 = -2	
**Publication Bias**	Undetected<br>Strongly suspected (-1)	only one study = 0	
**Other** (upgrading factors, circle all that apply)	Large effect (+1 or +2)<br>Dose response (+1 or +2)<br>No Plausible confounding (+1 or +2)	Remember RCT evidence quality is very rarely upgraded.	

QoL: low (-2)

**GRADE criteria**	**Rating** (circle one)	**Footnotes** (explain reasons for down- or upgrading)	**Quality of the evidence** (Circle one)
**Outcome:**			
**Study design**	RCT (starts as high quality)<br><br>Non-RCT (starts as low quality)	RCT = high	⊕⊕⊕⊕<br>High<br><br>⊕⊕⊕○<br>Moderate<br><br>⊕⊕○○<br>Low<br><br>⊕○○○<br>Very Low
**Risk of Bias** *(use the Cochrane Risk of Bias tables and figures)*	No<br>serious (-1)<br>very serious (-2)	very serious (2xsome concerns/5xhigh)= -2	
**Inconsistency**	No<br>serious (-1)<br>very serious (-2)	no heterogenity (5%) = No	
**Indirectness**	No<br>serious (-1)<br>very serious (-2)	indirectness does not appear to be an issue = No	
**Imprecision**	No<br>serious (-1)<br>very serious (-2)	nearly 400/ upper and lower confidence limit don´t cross SMD of 0.5 = No	
**Publication Bias**	Undetected<br>Strongly suspected (-1)	not all reaults from small studys/ funnel plot not evaluable = 0	
**Other** (upgrading factors, circle all that apply)	Large effect (+1 or +2)<br>Dose response (+1 or +2)<br>No Plausible confounding (+1 or +2)	Remember RCT evidence quality is very rarely upgraded.	
