# Supplementary material for: A systematic review and meta-analysis of the impact of relaxation techniques to reduce burden of disease in patients with psychotic disorders
Source: Sci Rep. 2026 Mar 24;16:9841. doi: 10.1038/s41598-026-44310-0 (PMC13018224; doi:10.1038/s41598-026-44310-0)
Supplement: Supplementary file 4 — Supplementary Material 4 [file 41598_2026_44310_MOESM4_ESM.docx]

**Supplementary Material, Forrest plots**

**A systematic review and meta-analysis of the impact of relaxation techniques to reduce burden of disease in patients with psychotic disorders**

Nina Schlößer^1^*, Christian Theisen^1^*, Eva Meisenzahl^1^, Carolin Kieckhäfer^1^

^1^ Department of Psychiatry and Psychotherapy, Medical Faculty, Heinrich-Heine University, Düsseldorf, Germany

*N. Schlösser and C. Theisen contributed equally to this work

Corresponding author: Christian Theisen, MD ([Christian.theisen@lvr.de](mailto:Christian.theisen@lvr.de))

Supplementary Figure 1, General psychotic symptoms. Subgroup Analysis Interventions, Forrest Plot

Supplementary Figure 2, Positive symptoms, subgroup Analysis Setting, Forrest plot

Supplementary Figure 3, Positive symptoms, subgroup Analysis Intervention, Forrest plot

Supplementary Figure 4, Negative symptoms, subgroup Analysis Setting, Forrest plot

Supplementary Figure 5, Negative symptoms, subgroup Analysis Intervention, Forrest plot

Supplementary Figure 6, Quality of life, subgroup Analysis setting, Forrest plot

Supplementary Figure 7, Quality of life, subgroup Analysis interventions, Forrest plot

Supplementary Figure 8, anxiety measurement, general meta-analysis, Forrest plot

Supplementary Figure 9, general psychotic symptoms, general meta-analysis, Forrest plot

Supplementary Figure 10, PANSS general, general meta-analysis, Forrest plot

Supplementary Figure 11, PANSS total, general meta-analysis, Forrest plot

Supplementary Figure 12, Positive symptoms, general meta-analysis, Forrest plot

Supplementary Figure 13, Negative symptoms, general meta-analysis, Forrest
